# Berberine ameliorates vascular dysfunction by a global modulation of lncRNA and mRNA expression profiles in hypertensive mouse aortae

**DOI:** 10.1371/journal.pone.0247621

**Published:** 2021-02-23

**Authors:** Na Tan, Yi Zhang, Yan Zhang, Li Li, Yi Zong, Wenwen Han, Limei Liu

**Affiliations:** 1 Department of Physiology and Pathophysiology, School of Basic Medical Sciences, Peking University, Beijing, China; 2 Key Laboratory of Molecular Cardiovascular Science, Ministry of Education, Beijing, China; The Chinese University of Hong Kong, HONG KONG

## Abstract

**Objective:**

The current study investigated the mechanism underlying the therapeutic effects of berberine in the vasculature in hypertension.

**Methods:**

Angiotensin II (Ang II)-loaded osmotic pumps were implanted in C57BL/6J mice with or without berberine administration. Mouse aortae were suspended in myograph for force measurement. Microarray technology were performed to analyze expression profiles of lncRNAs and mRNAs in the aortae. These dysregulated expressions were then validated by qRT-PCR. LncRNA-mRNA co-expression network was constructed to reveal the specific relationships.

**Results:**

Ang Ⅱ resulted in a significant increase in the blood pressure of mice, which was suppressed by berberine. The impaired endothelium-dependent aortic relaxation was restored in hypertensive mice. Microarray data revealed that 578 lncRNAs and 554 mRNAs were up-regulated, while 320 lncRNAs and 377 mRNAs were down-regulated in the aortae by Ang Ⅱ; both were reversed by berberine treatment. qRT-PCR validation results of differentially expressed genes (14 lncRNAs and 6 mRNAs) were completely consistent with the microarray data. GO analysis showed that these verified differentially expressed genes were significantly enriched in terms of “cellular process”, “biological regulation” and “regulation of biological process”, whilst KEGG analysis identified vascular function-related pathways including cAMP signaling pathway, cGMP-PKG signaling pathway, and calcium signaling pathway etc. Importantly, we observed that lncRNA ENSMUST00000144849, ENSMUST00000155383, and AK041185 were majorly expressed in endothelial cells.

**Conclusion:**

The present results suggest that the five lncRNAs ENSMUST00000144849, NR_028422, ENSMUST00000155383, AK041185, and uc.335+ might serve critical regulatory roles in hypertensive vasculature by targeting pivotal mRNAs and subsequently affecting vascular function-related pathways. Moreover, these lncRNAs were modulated by berberine, therefore providing the novel potential therapeutic targets of berberine in hypertension. Furthermore, lncRNA ENSMUST00000144849, ENSMUST00000155383, and AK041185 might be involved in the preservation of vascular endothelial cell function.

## Introduction

Hypertension is a leading common risk factor for worldwide morbidity and mortality from cardiovascular diseases [1–3], which is associated with the alterations in the function and structure of resistance and conduit arteries. Endothelial dysfunction (ED) may be either a cause or a consequence of hypertension. ED contributes to increased systemic vascular resistance and thus leads to the development and maintenance of hypertension [4]; persistent hypertension conversely impairs vascular endothelial function through reducing endothelial nitric oxide synthase (eNOS) activity or/and enhancing endoplasmic reticulum (ER) stress and oxidative stress [5–7].

Berberine, an alkaloid extract from many Chinese medicinal herbs, has long been used to treat gastrointestinal infections and diarrheas. In recent decades, multiple beneficial influences of berberine on cardiovascular system have been reported, including anti-hyperglycemic, anti-oxidative, cholesterol-lowering, and cardiac protective effects [8–10]. Both animal studies and clinical experiments indicate that berberine exerts protective effects on vascular endothelial function. Berberine attenuates carotid arterial endothelium-dependent contractions by inhibiting ER stress [11] and ameliorates aortic endothelial dysfunction [12] in spontaneously hypertensive rats (SHR), thus postponing the progress of hypertension. It improves flow-mediated vasodilation (FMD) possibly via reducing circulating endothelial microparticles in human [13]. Combination therapy with berberine and trimetazidine significantly increases FMD in patients with coronary heart disease and primary hypertension [14].

Long noncoding RNAs (lncRNAs), a class of noncoding RNAs, are endogenously transcripts with length of > 200 nucleotides [15]. Emerging evidence point out aspects on the relevance of lncRNAs to endothelial dysfunction in hypertension. LncRNA-GAS5 regulates endothelial cell function through β-catenin signaling in hypertension [16]. LncRNA-FENDRR mediates vascular endothelial growth factor to promote the apoptosis of brain microvascular endothelial cells in the intracerebral hemorrhage of hypertensive mice [17]. LncRNA-AK094457 accelerates angiotensin Ⅱ (Ang Ⅱ)-induced aortic endothelial dysfunction in SHR [18]. Berberine has been shown to play protective effects via regulating lncRNAs in colorectal cancer [19], myocardial hypertrophy [20], and ischemic brain [21]. However, whether berberine could affect the expressions of lncRNAs in endothelial cells in hypertension remains still unknown.

In the present study, we evaluated the benefit of berberine on aortic endothelium-dependent relaxation in Ang Ⅱ-mediated hypertensive mice. Furthermore, we performed systematical analyses to explore the regulatory effects of berberine on the lncRNA expressions in hypertensive mouse aortae and reveal the underlying molecular mechanisms. This study aimed at providing novel insights into the therapeutic role of berberine in hypertension and related cardiovascular diseases.

## Materials and methods

### Animal protocols

Male C57BL/6J mice (8–10 weeks old) were supplied by the Laboratory Animal Science Department of Peking University Health Science Center. Animal care and experimental procedures in this study were approved by the Animal Experimentation Ethics Committee of Peking University Health Science Center (LA2020122) and conformed to the Guide for the Care and Use of Laboratory Animals published by the US National Institute of Health (NIH Publication, 8th Edition, 2011).

Under ketamine/xylazine anesthesia (75 and 6 mg/kg), Ang Ⅱ (1mg/kg/day)- or PBS-loaded osmotic pumps were implanted in mice. Then the mice received berberine administration (100 mg/kg/day) or vehicle in drinking water for 2 weeks. Systolic blood pressure (SBP) was measured by the tail-cuff method before and after treatment for two weeks. Ang Ⅱ and berberine chloride were respectively purchased from Tocris Bioscience (Bristol, UK) and Sigma-Aldrich Chemical (St Louis, MO, USA). Ang Ⅱ was dissolved in PBS. Berberine was dissolved in drinking water.

### Artery preparation and functional assay

Mice were sacrificed by CO_2_ suffocation. Aortae from mice were removed and placed in ice-cold Krebs solution (mmol/L): 119 NaCl, 4.7 KCl, 1 MgCl_2_, 2.5 CaCl_2_, 1.2 KH_2_PO_4_, 25 NaHCO_3_, and 11 D-glucose. Then aortae were cleaned of adhering tissue and cut into segments of ~1.8 mm in length. Changes in isometric tension of aortic rings were recorded in myograph (Danish Myo Technology, Aarhus, Denmark). Each ring was stretched to 3 mN, stabilized for 90 min, and then contracted with 60 mmol/L KCl. After several washes by warmed Krebs solution, endothelium-dependent relaxations (EDRs) in response to acetylcholine (ACh, 0.003 to 10 μmol/L) were examined in the aortae pre-contracted with phenylephrine (Phe, 1 μmol/L). Followed by washes, the rings were subjected to 30-minute exposure to N^G^-nitro-_L_-arginine methyl ester (_L_-NAME, 100 μmol/L) and then contracted with Phe (1 μmol/L). Subsequently, endothelium-independent relaxations to cumulative additions of sodium nitroprusside (SNP, 0.001 to 10 μmol/L) were measured. Phe, ACh, _L_-NAME, and SNP were purchased from Sigma-Aldrich Chemical (St Louis, MO, USA) and dissolved in distilled water.

### Total RNA extraction

After mice were sacrificed, aortae were removed, cleaned of adhering tissue in sterilized PBS, and then stored in liquid nitrogen for total RNA extraction. Total RNA from each sample was extracted using Trizol reagent (Invitrogen Life Technologies, Carlsbad, CA, USA). RNA quantity and quality were measured by NanoDrop ND-1000 to make sure the high purity of the isolated RNA, as indicated by A260/280 ≥1.90 before microarray and quantitative real-time polymerase chain reaction (qRT-PCR) experiments.

### Microarray analysis

Microarray analysis was performed by KangChen Bio-tech (Shanghai, China). Briefly, extracted RNA samples was first purified and amplified. Then, the total RNA was transcribed into fluorescent cRNA along the entire length of the transcripts without 3′ bias utilizing a random priming method (Arraystar Flash RNA Labeling Kit, Arraystar). Finally, the labeled cRNA was hybridized onto the Arraystar Mouse LncRNA Array v3.0. After washing, the arrays were scanned on an Agilent Scanner G2505C. Agilent Feature Extraction software (version 11.0.1.1) was used to analyze the acquired array images. Quantile normalization and subsequent data processing were performed with the Gene Spring GX v12.1 software package (Agilent Technologies). Quantile-normalized lncRNAs and mRNAs with “Present” or “Marginal” (“All Targets Value”) flags were chosen for further data analysis. Differentially expressed lncRNAs and mRNAs were defined as fold change (the absolute ratio [no log scale] of normalized intensities between every two groups) >2.0 and p-value <0.05. The microarray data have been uploaded to NCBI Gene Expression Omnibus (GEO) under the accession number GSE159725 *(https://www.ncbi.nlm.nih.gov/geo/query/acc.cgi?acc=GSE159725)*.

### Quantitative real-time PCR validation

qRT-PCR was performed using SYBR^®^ High-Sensitivity qPCR SuperMix (NovoStart®, Shanghai, China) according to the manufacturer’s instruction. All experiments were conducted at least three times. The expression level of each gene was determined by the 2^−ΔΔCt^ method. The mRNA levels were normalized by GAPDH. All primers were shown in S1 Table (lncRNAs) and S2 Table (mRNAs).

### Functional annotation of differentially expressed lncRNAs and mRNAs

Gene ontology (GO) term enrichment analysis was used to elucidate the biological significance of the differentially expressed genes (DEGs), including cellular component, molecular function and biological process. Kyoto Encyclopedia of genes and genomes (KEGG) pathway analysis was performed to identify pathways in which DEGs significantly enriched. Differentially regulated mRNAs were uploaded into the Database for Annotation, Visualization and Integrated Discovery (DAVID, *https*:*//david*.*ncifcrf*.*gov/*) for annotation and functional analysis, including gene set enrichment analysis and mapping gene sets to the KEGG pathway [22]. The significant GO terms and pathways were determined using the Fisher’s exact test, and false discovery rate (FDR) was utilized to correct the p-values. p-value < 0.05 denotes statistical significance.

### lncRNA-mRNA co-expression network

A coding-non-coding gene co-expression network (CNC network) was constructed based on the correlation analysis between the differentially expressed lncRNAs and mRNAs [23]. LncRNAs and mRNAs with Pearson correlation coefficients not less than 0.9, p-value≤ 0.05, and FDR≤ 1 were selected to draw the network by the program of cytoscape. According to the size of the enrichment factor, the top 10 terms or pathways were extracted.

### Statistical analysis

All data were presented as mean ± SEM. One-way ANOVA followed by Tukey’s test and LSD for multiple comparisons were used in statistical analysis, which was performed in SPSS version 24.0 software. p-value < 0.05 was considered to be statistically significant.

A supplemental methods section can be found in the S1 File.

## Results

### Berberine lowers blood pressure and ameliorates aortic endothelial dysfunction in hypertensive mice

**Fig 1:**
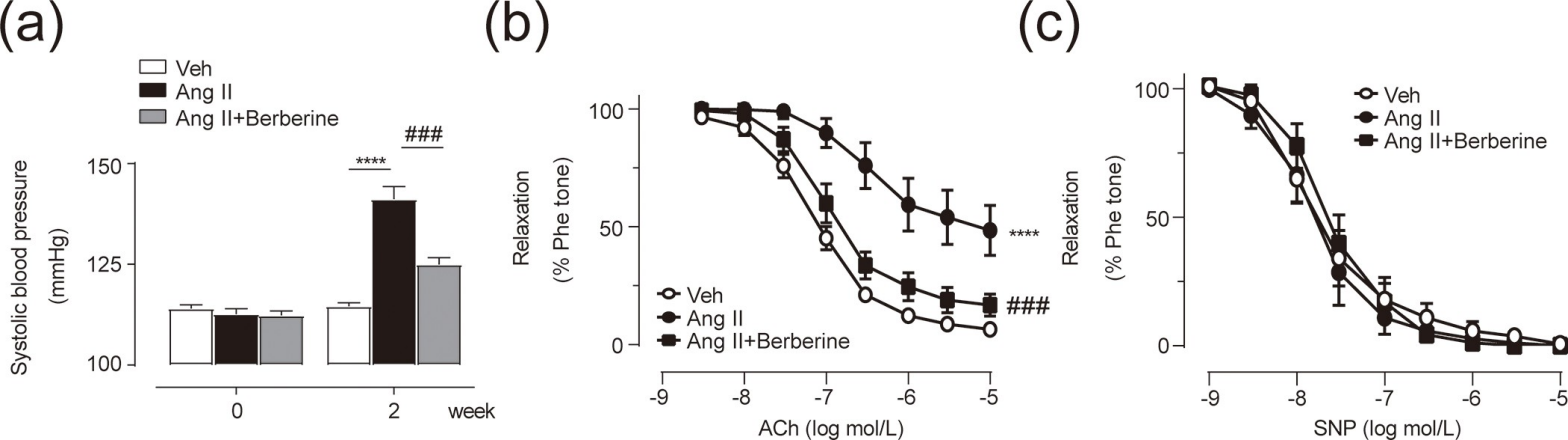
Berberine lowered Systolic Blood Pressure (SBP) and improved Endothelium-Dependent Relaxation (EDR) in the aortae of Ang Ⅱ-infused mice. (a) SBPs from Vehicle-, Ang Ⅱ- and Ang Ⅱ+Berberine-treated mice. (b) EDRs in the aortae from mice. (c) Endothelium-independent relaxations to sodium nitroprusside (SNP) in the mouse aortae. *p < 0.05, **p < 0.01, ***p < 0.001, ****p< 0.0001 *vs*. Veh, and #p < 0.05, ##p < 0.01, ###p < 0.001 *vs*. Ang Ⅱ. The bars indicate the standard error of the mean (n = 5 for SBP; n = 4 for EDRs; n = 4 for SNP-induced relaxations). Veh, vehicle; Ang Ⅱ, angiotensin Ⅱ.

To confirm the therapeutic effect of berberine on hypertensive vasculature, we induced hypertension in mice with Ang Ⅱ. As shown in Fig 1A, 2-week infusion with Ang Ⅱ induced the marked increase of systolic blood pressure (SBP) in mice and co-treatment with berberine significantly lowered the SBP in hypertensive mice. Ang Ⅱ-infused mice exhibited the impaired EDRs to ACh in the aortae, which were restored by berberine administration (Fig 1B). By contrast, endothelium-independent relaxations to SNP were similar among all the groups (Fig 1C). These data suggest that berberine ameliorated endothelial dysfunction in the conduit vessels in Ang Ⅱ-mediated hypertension, thus attenuating the progression of hypertension.

### Microarray analysis for gene expression profiles

**Fig 2:**
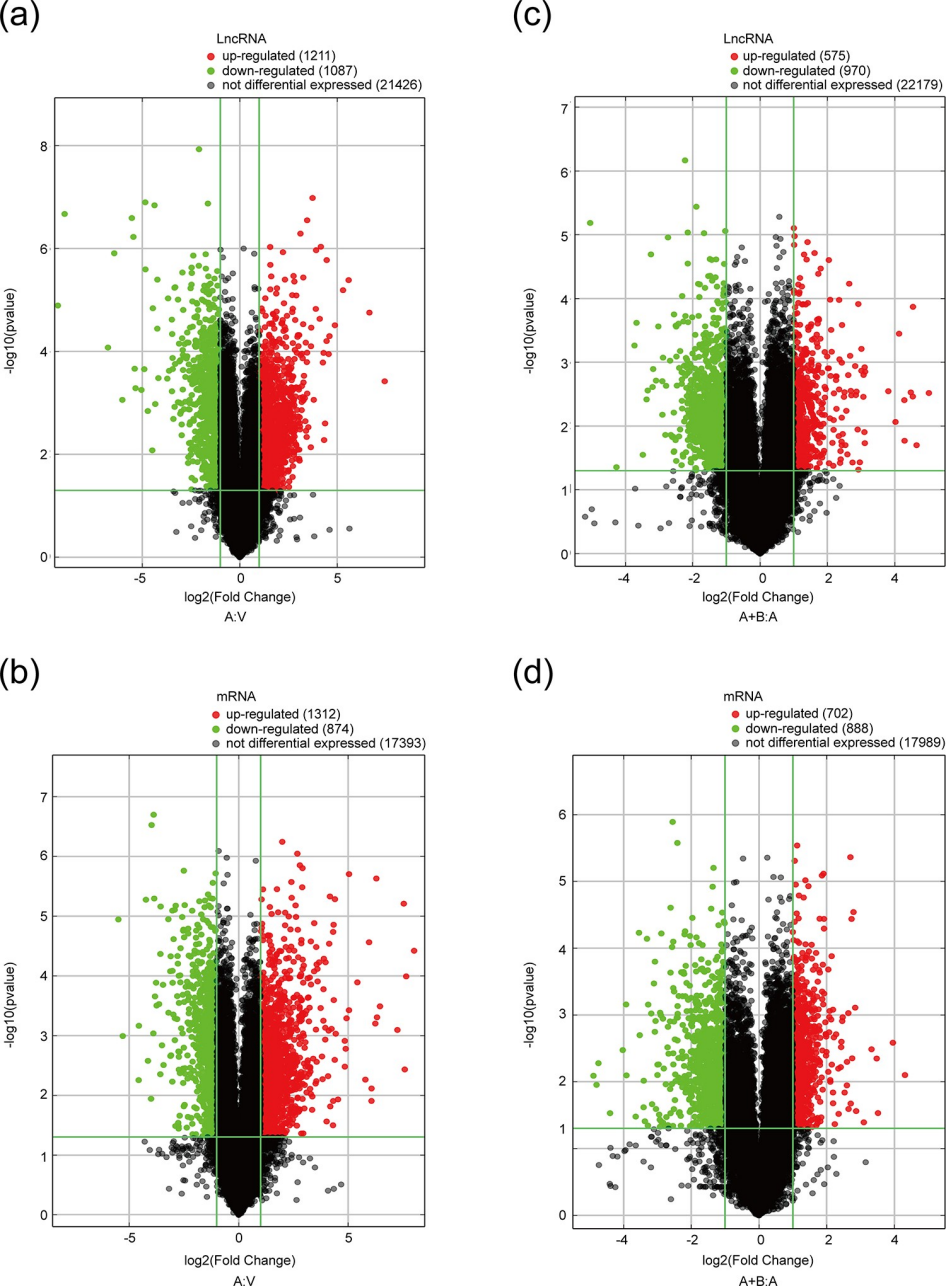
Identification of the Differentially Expressed (DE) lncRNAs and mRNAs. The Volcano plot analysis showed DE-lncRNAs in Ang Ⅱ-treated group compared with Vehicle-treated group (a) and DE-lncRNAs in Ang Ⅱ+Berberine co-treated group compared with Ang Ⅱ-treated group (c); volcano plot identified the dysregulated mRNAs between Ang Ⅱ and Vehicle groups (b) and Ang Ⅱ+Berberine and Ang Ⅱ groups (d). Red and green represented the up-regulated and down-regulated lncRNAs or mRNAs respectively; the vertical lines corresponded to 2-fold up-regulation or down-regulation, and the horizontal lines represented p = 0.05. A: V, Ang Ⅱ (angiotensin Ⅱ) *vs*. Vehicle. A+B: A, Ang Ⅱ+Berberine *vs*. Ang Ⅱ.

To identify possible lncRNAs and mRNAs participated in the efficacy of berberine, we performed a microarray-based analysis of gene expression profiles in the three groups of mice. By comparison of Ang Ⅱ-treated group and Vehicle-treated group, we identified 2298 differentially expressed (DE) lncRNAs (including 1211 up-regulated and 1087 down-regulated, Fig 2A) and 2186 DE-mRNAs (including 1312 up-regulated and 874 down-regulated, Fig 2B). The expression levels of lncRNAs and mRNAs were dysregulated through over-expression or under-expression. Among 1545 DE-lncRNAs, 575 lncRNAs were up-regulated and 970 lncRNAs were down-regulated in Ang Ⅱ+Berberine-treated group compared with Ang Ⅱ-treated group (Fig 2C). Among 1590 DE-mRNAs, 702 mRNAs were significantly increased whereas 888 mRNAs were remarkably decreased in Ang Ⅱ+Berberine-treated group (Fig 2D). Distinguishable expression patterns of top 60 lncRNAs and mRNAs were presented as the heat maps of hierarchical clustering in S1 Fig. This part of the results implicates that berberine might improve vascular function through regulating expressions of lncRNAs and mRNAs in hypertension.

**Fig 3:**
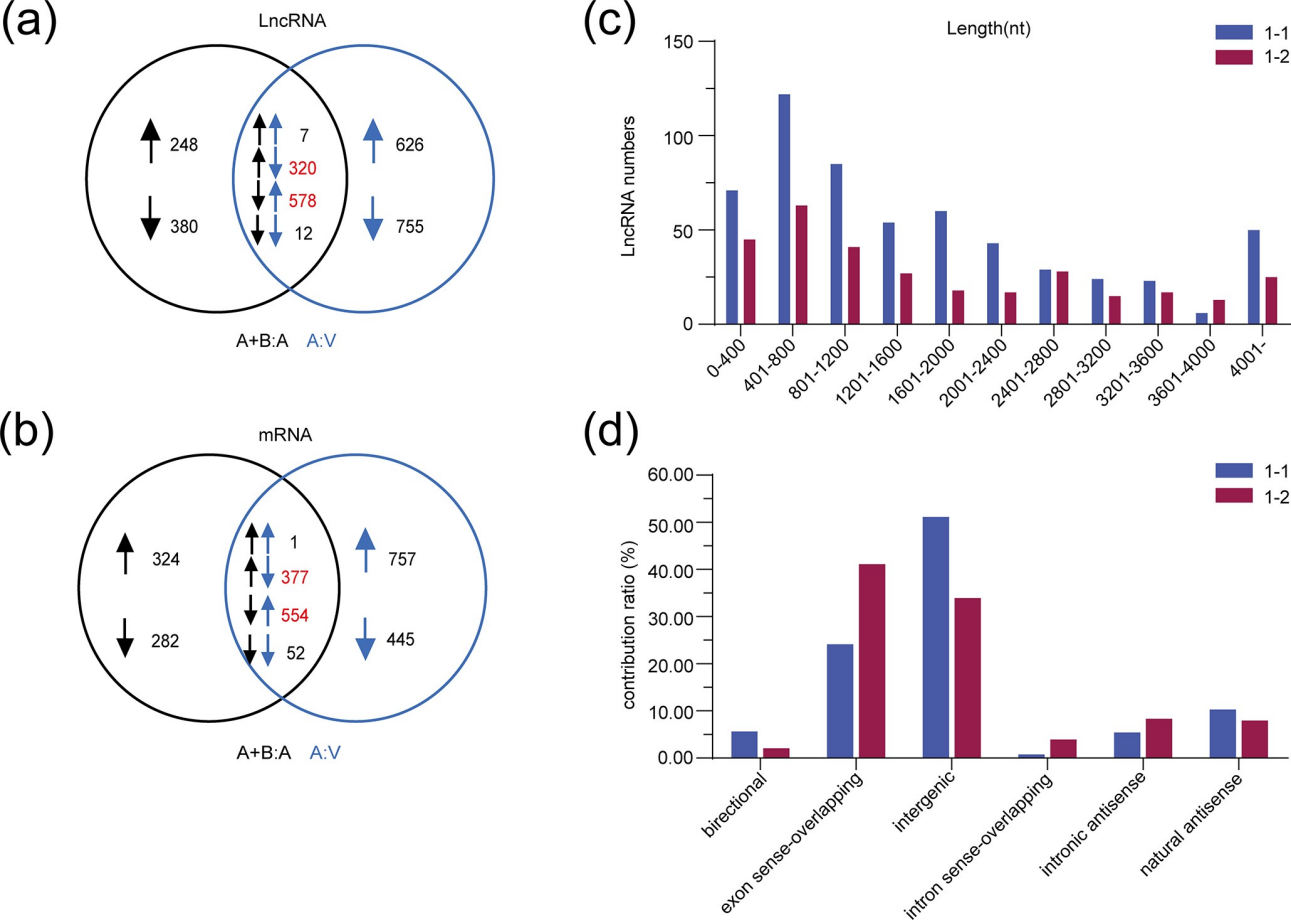
The description of the microarray analysis. Venn diagrams of differentially expressed (DE) lncRNAs (a) and DE-mRNAs (b) in the mouse aortae. The black or blue arrow represented the comparison of Ang Ⅱ+Berberine and Ang Ⅱ groups or Ang Ⅱ and Vehicle groups. The up-arrows indicated up-regulation and the down-arrows indicated down-regulation. (c) The DE-lncRNAs were mainly between 400 and 1200 bp in length. (d) The relationship between these dysregulated lncRNAs and their targets. A: V, Ang Ⅱ *vs*. Vehicle. A+B: A, Ang Ⅱ+Berberine *vs*. Ang Ⅱ; 1–1, genes up-regulated by Ang Ⅱ but down-regulated by co-treatment with berberine; 1–2, genes suppressed by Ang Ⅱ whilst reversed by berberine; Ang Ⅱ, angiotensin Ⅱ.

In order to further understand the regulatory effects of berberine on gene expression in hypertensive vessels, the Venn diagram was performed to overlap the genes. Among the overlapped genes, the 320 decreased lncRNAs and 377 down-regulated mRNAs by Ang Ⅱ were rescued after berberine administration; while berberine suppressed the up-regulated expressions of 578 lncRNAs and 554 mRNAs in hypertensive mouse aortae (Fig 3A and 3B). In order to have insight into the expression pattern of these dysregulated lncRNAs, we first divided the overlapped lncRNAs into two categories: 1–1 and 1–2 (1–1, genes up-regulated by Ang Ⅱ but down-regulated by co-treatment with berberine; 1–2, genes suppressed by Ang Ⅱ while rescued by berberine). The general signatures of these lncRNAs, including length distribution, classification, and chromosomal distribution, were thus summarized. These lncRNAs mainly ranged from 400 to 1200 bp in length (Fig 3C). In addition, Fig 3D revealed that these dysregulated lncRNAs mainly fell into six categories including exon sense-overlapping, intergenic, natural antisense, intronic antisense, bidirectional, and intron sense-overlapping. Moreover, more than 70% of the dysregulated lncRNAs were exon sense-overlapping and intergenic lncRNAs. In addition, we report that berberine-regulated lncRNAs were located at human chromosomes 1–19, X and Y. The under-expressed lncRNAs by berberine mostly located on 2 and 4 chromosomes (S2A Fig), whereas the elevated lncRNAs by berberine were mainly located at chromosome 2 and 11 (S2B Fig).

### GO analysis and KEGG pathway enrichment analysis

To analyze the functional enrichment and related signaling pathways of DEGs regulated by berberine in hypertension, the Gene Ontology (GO) along with the Kyoto Encyclopedia of Genes and Genomes (KEGG) were performed. The gene ontologies cover three domains including biological process (BP), cellular component (CC), and molecular function (MF). The top 10 significantly enriched GO terms in each domain were respectively shown in S3 and S4 Figs. In category 1–1 (S3 Fig), the meaningful BP terms are associated with immune system process (GO:0002376) and cell chemotaxis (GO:0060326); the DEGs of CC were obviously enriched in cell periphery (GO:0071944), plasma membrane (GO:0005886), and extracellular region (GO:0005576). In category 1–2 (S4 Fig), the DEGs were most significantly enriched in cellular process (GO:0009987) in the BP category; the DEGs under the CC were related to cell (GO:0005623) and cell part (GO:0044464). In addition, the terms in MF suggested that protein binding (GO:0005515) and binding (GO:0005488) seemed particularly important in the improvement of vascular function by berberine (S3 and S4 Figs). KEGG pathway analysis revealed that Ang Ⅱ might impair vascular function via up-regulating cytokine-cytokine receptor interaction, chemokine signaling, and cell adhesion molecules (S5A Fig) and suppressing cGMP-PKG signaling, vascular smooth muscle contraction, and ECM-receptor (S6A Fig). However, PPAR signaling, cytokine-cytokine receptor interaction, PI3K-Akt signaling, vascular smooth muscle contraction, and ECM-receptor were strongly influenced by berberine. The first three pathways (S5B Fig) were attenuated and the final two pathways (S6B Fig) were stimulated by berberine, thus improving vascular function in hypertension.

### qRT-PCR validation

**Fig 4:**
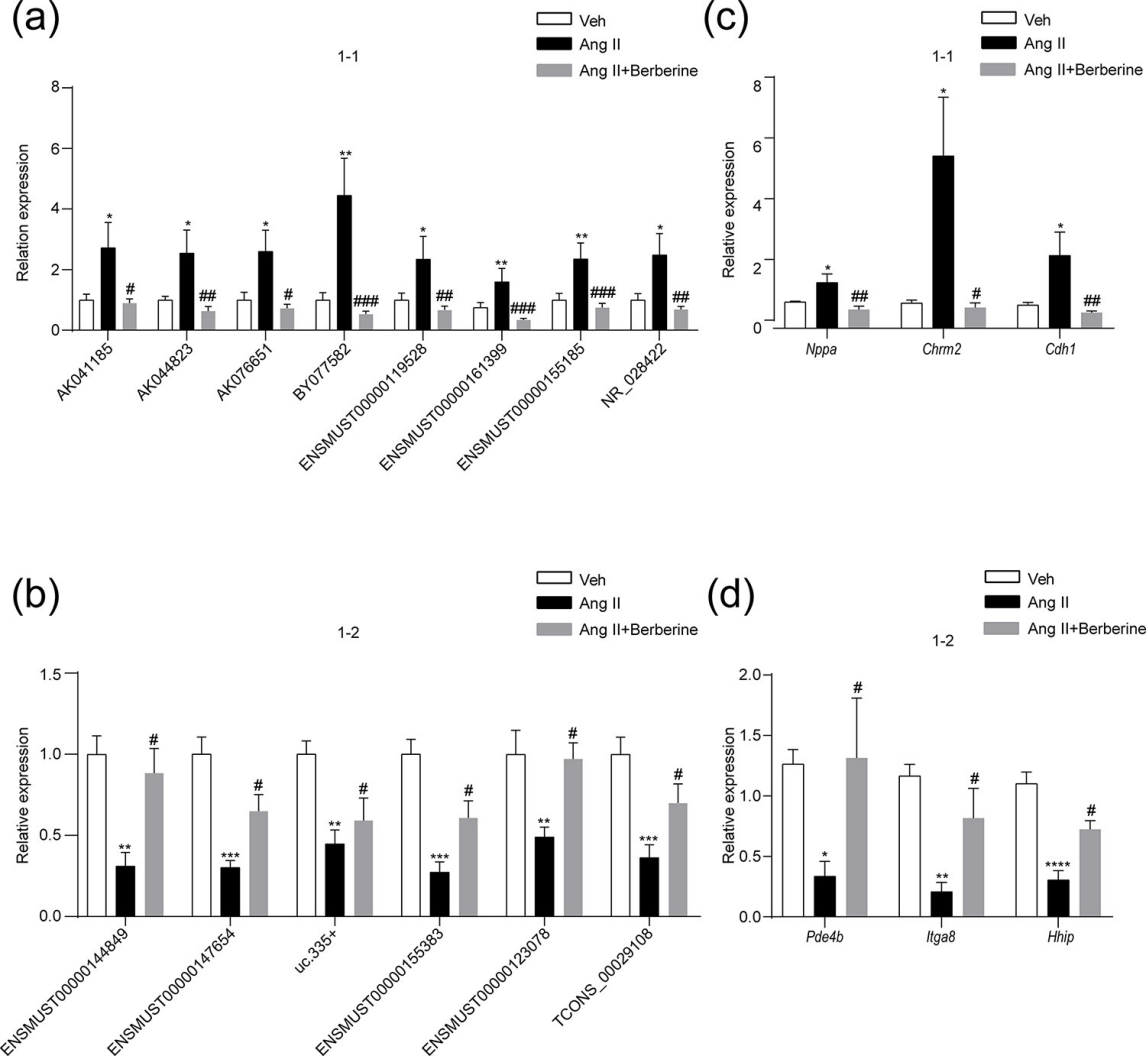
Validation of DE-lncRNAs and mRNAs. Quantitative RT-PCR demonstrated the dysregulated lncRNAs including 8 up-regulated DE-lncRNAs (a) and 6 down-regulated DE-lncRNAs (b) induced by Ang Ⅱ in the aortae from mice; the dysregulated expressions of lncRNAs were modified by berberine administration (a, b). qRT-PCR validation results showed that 3 up-regulated (c) and 3 down-regulated (d) DE-mRNAs caused by Ang Ⅱ were also modulated by berberine in hypertensive mouse aortae (c, d). *p < 0.05, **p < 0.01, ***p < 0.001, ****p< 0.0001 *vs*. Vehicle, and #p < 0.05, ##p < 0.01, ###p < 0.001 *vs*. Ang Ⅱ. The bars indicate the standard error of the mean (n = 5). 1–1, genes up-regulated by Ang Ⅱ but down-regulated by berberine; 1–2, genes suppressed by Ang Ⅱ while increased by berberine; Veh, vehicle; Ang Ⅱ, angiotensin Ⅱ.

To validate the dysregulated expressions of lncRNAs and mRNAs derived from microarray data, we performed Quantitative real-time polymerase chain reaction (qRT-PCR). 14 dysregulated lncRNAs, including 8 up-regulated DE-lncRNAs (Fig 4A: AK041185, AK044823, AK076651, BY077582, ENSMUST00000119528, ENSMUST00000161399, ENSMUST00000155185, and NR_028422) and 6 down-regulated DE-lncRNAs (Fig 4B: ENSMUST00000144849, ENSMUST00000147654, uc.335+, ENSMUST00000155383, ENSMUST00000123078, and TCONS_00029108) induced by Ang Ⅱ, were successfully verified by qRT-PCR. Furthermore, these dysregulated expressions of lncRNAs were modified by berberine administration (Fig 4A and 4B). In the meanwhile, 3 up-regulated (*Nppa*, *Chrm2*, *and Cdh1*) and 3 down-regulated (*Pde4b*, *Itga8*, *Hhip*) DE-mRNAs by the induction of Ang Ⅱ were selected for qRT-PCR validation. The validation results also revealed that berberine rescued the dysregulation of these DE-mRNAs in hypertensive vasculature (Fig 4C and 4D). The expression patterns of these lncRNAs and mRNAs in qRT-PCR validation results were consistent with those in microarray results. To sum up, these genes may play critical roles in the anti-hypertensive effect of berberine (the detailed information of the 14 lncRNAs and the 6 mRNAs was shown in the S3 and S4 Tables respectively).

### Construction of DE-lncRNA-mRNA co-expression network

**Fig 5:**
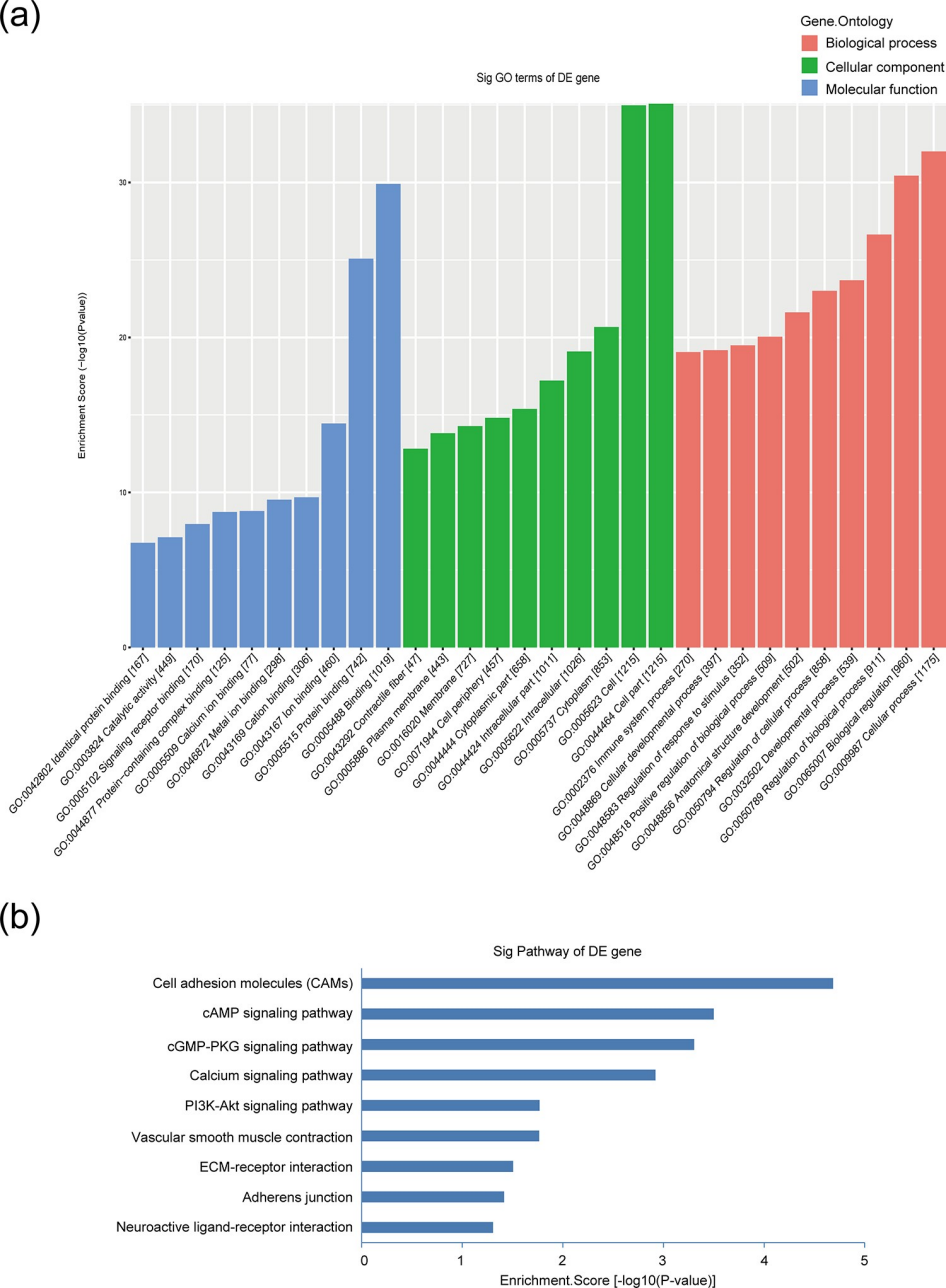
GO and pathway analysis of the dysregulated lncRNAs-co-expressed mRNAs. (a) The top 10 GO terms of biological process (blue), cellular components (green) and molecular functions (red) were listed. (b) The 9 pathways in which these 14 DE-lncRNAs were enriched.

**Fig 6:**
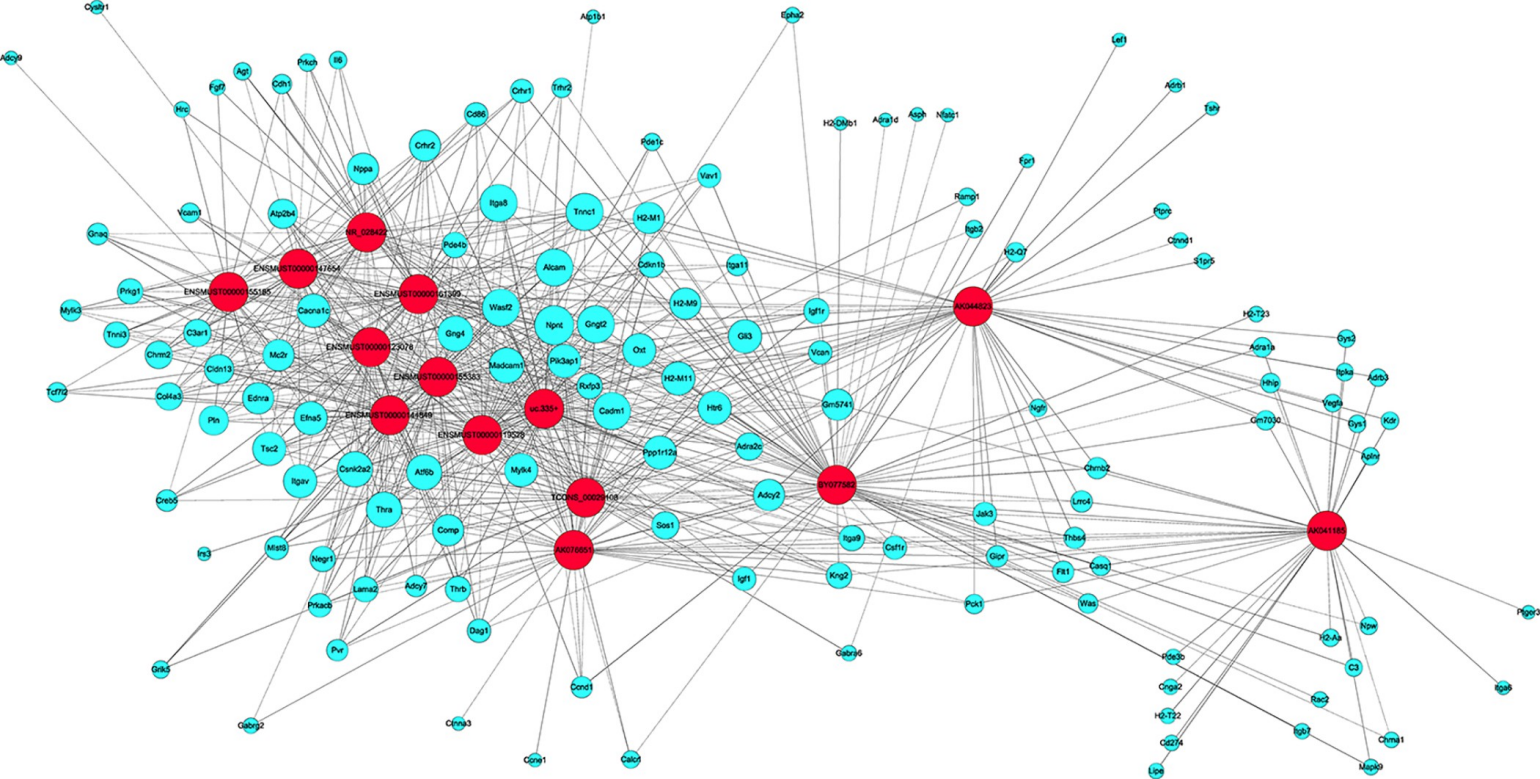
The lncRNA-mRNA co-expression network. It included 14 differentially expressed lncRNAs and 137 most highly relevant dysregulated mRNAs including the 6 mRNAs validated by qRT-PCR. Blue nodes were the coding genes; red nodes were lncRNAs. The solid line indicated a positive correlation and the dotted line indicated a negative correlation.

To reveal the potential roles of hypertension-related lncRNAs and mRNAs, the DE-lncRNA-mRNA co-expression network was constructed. On the basis of lncRNA-mRNA co-expression network and genomic co-location, the roles of lncRNAs and mRNAs in the improvement of vascular function by berberine in hypertension were also predicted. GO enrichment indicated that the 14 DE-lncRNAs were involved in multiple molecular functions, such as “cellular process (GO:0009987)”, “biological regulation (GO:0065007)” and “regulation of biological process (GO:0050789)” (Fig 5A). According to the KEGG pathway analysis, these DE-lncRNAs were enriched in the pathways of cell adhesion molecules, cAMP signaling pathway, cGMP-PKG signaling pathway, calcium signaling pathway, PI3K-Akt signaling pathway, vascular smooth muscle contraction, ECM-receptor interaction, adherens junction, and neuroactive ligand-receptor interaction, which were all associated with vascular function (Fig 5B). Based on the expression profiling, the significantly DE-mRNAs involved in these pathways were selected to construct the lncRNA-mRNA co-expression network; the latter included 14 aberrantly expressed lncRNAs and 137 most highly relevant dysregulated mRNAs including the 6 validated mRNAs (Fig 6).

## Discussion

Berberine and its derivatives have been shown to attenuate the development of hypertension by ameliorating vascular dysfunction [11, 12, 24]. The current study strengthens the evidence that berberine protects vascular endothelial function in hypertensive mice. However, the molecular mechanisms underlying the beneficial effects of berberine on hypertensive vasculature remained to be further explored. Therefore, we performed the microarray analysis to identify differentially expressed (DE) genes in the aortae from Ang Ⅱ-induced hypertensive mice under the treatment with vehicle or berberine. We observed 2298 DE-lncRNAs and 2186 DE-mRNAs in hypertensive mice. Moreover, 1545 DE-lncRNAs and 1590 DE-mRNAs were identified in hypertensive mice after berberine treatment. We for the first time implicated that berberine might improve vascular function through regulating expressions of lncRNAs in hypertension.

LncRNAs, a novel class of non-coding RNAs, play critical roles at transcriptional and post-transcriptional levels in a variety of physiological and pathological processes [25]. In recent years, lncRNAs are emerged as novel modulators in the development of hypertension [18, 26, 27]. LncRNA TUG1 promotes the proliferation and migration of vascular smooth muscle cells (VSMCs) by affecting miR-145-5p/FGF10 axis in hypertensive state [28]. Here, we revealed that 1211 lncRNAs were up-regulated and 1087 were down-regulated among 2298 DE-lncRNAs in hypertensive mouse aortae, suggesting the vital roles of lncRNAs in the occurrence and development of vascular dysfunction in hypertension. Berberine has been reported to ameliorate nonalcoholic fatty liver disease by modulating hepatic lncRNA expression profiles and reverse down-regulated MRAK052686 [29]. Although a good deal of evidence suggests the relevance of lncRNAs to endothelial dysfunction in hypertension, it is still unclear whether berberine improves vascular endothelial function by modulating lncRNA expressions in hypertension. Our microarray data observed 970 down-regulated and 575 up-regulated lncRNAs in hypertensive mice with the therapy of berberine.

Summing up the results of existing studies, we can learn that several proteins are involved in regulating the progression of high blood pressure, such as endothelin-1, Rac1, and G protein-coupled estrogen receptor [30–32], but hypertension-related genes have not been identified systemically. Here, we revealed that 2186 DE-mRNAs (including 1312 up-regulated mRNAs and 874 down-regulated mRNAs) were discovered in hypertensive mice. Simultaneously, 1590 DE-mRNAs were identified in berberine-treated hypertensive mice; among them were 702 elevated and 888 suppressed mRNAs. Next, the current study focused on the exploration of lncRNAs and mRNAs involved in the treatment with berberine to vascular function in hypertension. The GO analysis showed that 14 DE-lncRNAs (AK041185, AK044823, AK076651, BY077582, ENSMUST00000119528, ENSMUST00000161399, ENSMUST00000155185, NR_028422, ENSMUST00000144849, ENSMUST00000147654, uc.335+, ENSMUST00000155383, ENSMUST00000123078, and TCONS_00029108) were mainly involved in molecular functions, including biological regulation, cellular process, and regulation of biological process, which might be closely related to the regulation of vascular function by berberine. However, most of these lncRNAs were never reported both in the progress of hypertension and in the regulation of berberine on hypertensive vasculature. Importantly, we obtained the qRT-PCR validation results which were consistent with microarray results. This study comprehensively revealed the lncRNAs related to vascular dysfunction in hypertension. In addition, we provided a new perspective for the treatment of berberine on vascular dysfunction in hypertension.

Recent studies show that lncRNA-mRNA co-expression network analysis is widely used to evaluate the functional roles of lncRNAs, thus assessing the relationship between the lncRNA expressions and the cardiovascular diseases. For example, Zhang *et al*. performed lncRNA-mRNA co-expression analysis in atherosclerosis [33] and Pant *et al*. constructed lncRNAs and mRNAs mediated co-expression network in diabetic cardiomyopathy [34]. Here, we also performed co-expression network analysis of the 14 DE-lncRNAs and the network consisted of 720 specific lncRNA-mRNA co-expression relationships. Interestingly, we observed these lncRNAs co-expressed with 137 sense mRNAs and were involved in many pathways, including cell adhesion molecules (CAMs), cAMP signaling pathway, cGMP-PKG signaling pathway, calcium signaling pathway, PI3K-Akt signaling pathway, vascular smooth muscle contraction, ECM-receptor interaction, adherens junction, and neuroactive ligand-receptor interaction, which were closely associated with vascular function. qRT-PCR validation also verified that the up-regulated (*Nppa*, *Chrm2*, and *Cdh1*) and the down-regulated (*Pde4b*, *Itga8*, *Hhip*) DE-mRNAs in the aortae from hypertensive mice were reversed by berberine; these data were consistent with the microarray data. Just as important, these 6 validated DE-mRNAs were included in the co-expression network. CAMs and ECM-receptor interaction signaling pathways are mainly involved in cell adhesion [35]. We found that the genes in the enrichments included the DE genes involved with the cadherin and integrin family (*Cdh1* and *Itga8*). According to our microassay results, *Cdh1* was co-expressed with lncRNA NR_028422 and *Itga8* was co-expressed with lncRNA ENSMUST00000155383 with extremely positively correlation; we also noticed that *Itga8* was also involved in PI3K-Akt signaling pathway. Except for natriuretic and diuretic effects, atrial natriuretic peptide (ANP) has been reported to elicit vasorelaxant action, thereby reducing body fluid volume and maintaining blood pressure homeostasis [36]. *Nppa* (encoding pro-ANP) has been shown to play a critical role in the reduction of SBP in hypertensive rats [37, 38]. Our current study announced that *Nppa* was elevated in hypertensive mouse aortae but attenuated by berberine, which might be closely related to lncRNA ENSMUST00000144849. Phosphodiesterases (PDE) limits the effects of vasodilators such as nitric oxide (NO). *Pde4b* hydrolyzes cyclic-AMP (cAMP); the latter suppresses PI3K/Akt signals and then restrains the vessel formation [39]. The expression of *Pde4b* was down-modulated in SHR preglomerular microvascular smooth muscle cells (SMCs) and endothelial cells (ECs) [40], but its expression can be induced by NO in rat pulmonary artery SMCs [41]. Our results also showed the reduced level of *Pde4b* in hypertension and the reversal effect of berberine on its expression. Furthermore, lncRNA uc.335+ might participate in regulating *Pde4b* in the therapy of berberine to hypertensive arterial function. *Chrm2*, associated with cAMP signaling pathway, has been reported to increase arterial contraction and lead to an increase of blood pressure [42]. We presented here an up-regulation of *Chrm2* and the inhibitory effect of berberine to *Chrm2* in the hypertensive mice. What’s more, it was probably regulated by lncRNA NR_028422. *Hhip* gene, highly expressed in ECs, has a crucial role in controlling angiogenesis [43], yet the protective role of *Hhip* in the endothelial injury under hypertension is not understood. Both microarray analysis and qRT-PCR validation proved the under-expression of *Hhip* in hypertensive mouse aortae. Furthermore, the *Hhip* level was rescued and lncRNA AK041185 is possibly involved in the regulation of *Hhip* under the action of berberine. In order to explore whether the 5 lncRNAs were involved in the improvement of endothelial function in hypertension, we examined the expressions of the 5 lncRNAs both in ECs and in SMCs from C57BL/6J mouse aortae. As shown in S7 Fig, the levels of lncRNA ENSMUST00000144849, ENSMUST00000155383, and AK041185 were significantly higher in ECs than those in SMCs. LncRNA uc.335+ and lncRNA NR_028422 were also clearly expressed in ECs; but the expression level of lncRNA uc.335+ was similar in ECs to that in SMCs and the expression level of lncRNA NR_028422 in SMCs was obviously higher than that in ECs. Based on these observations, we speculated the potential benefits of ENSMUST00000155383, AK041185, and ENSMUST00000144849 in the maintenance of endothelial function.

## Conclusion

Taken together, these findings indicate that lncRNA ENSMUST00000144849, NR_028422, ENSMUST00000155383, AK041185, and uc.335+ may play critical roles by modulating the relevant mRNAs in the remedy of berberine to hypertensive vascular damage. This study provides novel insights of how lncRNAs affect hypertension and which pathways may play important roles during the condition. These results could help understand the biological mechanisms of berberine on the improvement of vascular function and structure in treating hypertension.

## Limitations

Our study revealed the possibility that lncRNA ENSMUST00000144849, ENSMUST00000155383, and AK041185 participated in vascular dysfunction in hypertension and could be regulated by berberine. Although we verified that they were majorly expressed in ECs, we did not make it clear whether the 3 lncRNAs were exactly involved in the improvement of vascular endothelial function in hypertension. Therefore, we should explore the expressions of these 3 lncRNAs in endothelial cells (ECs) from hypertensive mice and the regulatory effects of berberine on them in ECs under hypertensive state. In addition, the biology functions of lncRNA ENSMUST00000144849, ENSMUST00000155383, and AK041185 ought to be investigated by overexpression or knockdown of the lncRNAs, thus proving the correlation about the changes of the 3 lncRNAs and related mRNAs with the berberine treatment in hypertension. However, this is beyond the scope of the present study.

## Supporting information

S1 Table The primers of the lncRNAs. (DOCX)

S2 Table The primers of the mRNAs. (DOCX)

S3 Table Functional annotation of differentially expressed lncRNAs. (DOCX)

S4 Table Functional annotation of differentially expressed mRNAs. (DOCX)

S1 File Supplemental methods section. (DOCX)

S1 Fig Hierarchical clustering analysis. It showed top 60 differentially expressed lncRNAs (a) and mRNAs (b) among the three groups. Red color indicates highly relative expression and green color indicates low relative expression. V, Vehicle; A, Ang Ⅱ, angiotensin Ⅱ; B, Ang Ⅱ+Berberine.
(TIF)

S2 Fig Chromosomal distribution of the 1–1 (a) and 1–2 (b) lncRNAs. 1–1, genes up-regulated by Ang Ⅱ but down-regulated by co-treatment with berberine; 1–2, genes suppressed by Ang Ⅱ while reversed by berberine. Ang Ⅱ, angiotensin Ⅱ.
(TIF)

S3 Fig GO enrichment analysis of category 1–1. Red, green and blue bars represented biological process (BP), cellular component (CC), and molecular function (MF). (a) The top 10 GO terms that were associated with the coding gene function of up-regulated lncRNAs in the Ang Ⅱ-treated group compared with Vehicle-treated group. (b) the top 10 GO terms that were associated with the coding gene function of down-regulated lncRNAs in the Ang Ⅱ+Berberine co-treated group compared with Ang Ⅱ-treated group. 1–1, genes up-regulated by Ang Ⅱ but down-regulated by berberine; V, Vehicle; A, Ang Ⅱ, angiotensin Ⅱ; A+B, Ang Ⅱ+Berberine.
(TIF)

S4 Fig GO enrichment analysis of category 1–2. Red, green and blue bars represented biological process (BP), cellular component (CC), and molecular function (MF). (a) The top 10 GO terms that were associated with the coding gene function of down-regulated lncRNAs in the Ang Ⅱ-treated group compared with Vehicle-treated group. (b) the top 10 GO terms that were associated with the coding gene function of up-regulated lncRNAs in the Ang Ⅱ+Berberine co-treated group compared with Ang Ⅱ-treated group. 1–2, genes suppressed by Ang Ⅱ while increased by berberine; V, Vehicle; A, Ang Ⅱ, angiotensin Ⅱ; A+B, Ang Ⅱ+Berberine.
(TIF)

S5 Fig KEGG pathway analysis of 1–1. It revealed that (a) the top 10 pathways that were associated with the coding gene function of up-regulated lncRNAs in the Ang Ⅱ-treated group compared with Vehicle-treated group, (b) the top 10 pathways that were associated with the coding gene function of down-regulated lncRNAs in the Ang Ⅱ+Berberine co-treated group compared with Ang Ⅱ-treated group. 1–1, genes up-regulated by Ang Ⅱ but down-regulated by berberine; V, Vehicle; A, Ang Ⅱ, angiotensin Ⅱ; A+B, Ang Ⅱ+Berberine.
(TIF)

S6 Fig KEGG pathway analysis of 1–2. It showed (a) the top 10 pathways that were associated with the coding gene function of down-regulated lncRNAs in the Ang Ⅱ-treated group compared with Vehicle-treated group, (b) the top 10 pathways that were associated with the coding gene function of up-regulated lncRNAs in the Ang Ⅱ+Berberine co-treated group compared with Ang Ⅱ-treated group. 1–2, genes suppressed by Ang Ⅱ while increased by berberine; V, Vehicle; A, Ang Ⅱ, angiotensin Ⅱ; A+B, Ang Ⅱ+Berberine.
(PNG)

S7 Fig The expressions of the five lncRNAs in primary mouse aortic ECs and SMCs. LncRNA ENSMUST00000144849, lncRNA ENSMUST00000155383, lncRNA AK041185, and lncRNA NR_028422 exhibited different expression levels in ECs and SMCs; but lncRNA uc.335+ expression was similar in the two cell types. EC, endothelial cells; SMCs, smooth muscle cells.
(TIF)

## References

[1] Dinh QN, Drummond GR, Sobey CG, Chrissobolis S. Roles of inflammation, oxidative stress, and vascular dysfunction in hypertension. Biomed Res Int. 2014;2014:406960 10.1155/2014/406960 . 25136585 PMC4124649

[2] Fan WG, Xie F, Wan YR, Campbell NRC, Su H. The impact of changes in population blood pressure on hypertension prevalence and control in China. J Clin Hypertens. 2020;22(2):150–6. 10.1111/jch.13820 . 32003937 PMC8030006

[3] GBD 2015 Risk Factors Collaborators. Global, regional, and national comparative risk assessment of 79 behavioural, environmental and occupational, and metabolic risks or clusters of risks, 1990–2015: a systematic analysis for the Global Burden of Disease Study 2015. Lancet. 2016;388(10053):1659–724. 10.1016/S0140-6736(16)31679-8 . 27733284 PMC5388856

[4] Chrissobolis S, Faraci FM. The role of oxidative stress and NADPH oxidase in cerebrovascular disease. Trends Mol Med. 2008;14(11):495–502. 10.1016/j.molmed.2008.09.003 . 18929509 PMC3140460

[5] Kassan M, Galán M, Partyka M, Saifudeen Z, Henrion D, Trebak M, et al Endoplasmic reticulum stress is involved in cardiac damage and vascular endothelial dysfunction in hypertensive mice. Arterioscler Thromb Vasc Biol. 2012;32(7):1652–61. 10.1161/ATVBAHA.112.249318 . 22539597 PMC5374512

[6] Galán M, Kassan M, Kadowitz PJ, Trebak M, Belmadani S, Matrougui K. Mechanism of endoplasmic reticulum stress-induced vascular endothelial dysfunction. Biochim Biophys Acta. 2014;1843(6):1063–75. 10.1016/j.bbamcr.2014.02.009 . 24576409 PMC4086191

[7] Dharmashankar K, Widlansky ME. Vascular endothelial function and hypertension: insights and directions. Curr Hypertens Rep. 2010;12(6):448–55. 10.1007/s11906-010-0150-2 . 20857237 PMC2982873

[8] Cheng F, Wang Y, Li J, Su C, Wu F, Xia WH, et al Berberine improves endothelial function by reducing endothelial microparticles-mediated oxidative stress in humans. Int J Cardiol. 2013;167(3):936–42. 10.1016/j.ijcard.2012.03.090 . 22465347

[9] Lee S, Lim HJ, Park HY, Lee KS, Park JH, Jang Y. Berberine inhibits rat vascular smooth muscle cell proliferation and migration in vitro and improves neointima formation after balloon injury in vivo. Berberine improves neointima formation in a rat model. Atherosclerosis. 2006;186(1):29–37. 10.1016/j.atherosclerosis.2005.06.048 . 16098530

[10] Wang Y, Huang Y, Lam KS, Li Y, Wong WT, Ye H, et al Berberine prevents hyperglycemia-induced endothelial injury and enhances vasodilatation via adenosine monophosphate-activated protein kinase and endothelial nitric oxide synthase. Cardiovasc Res. 2009;82(3):484–92. 10.1093/cvr/cvp078 . 19251722

[11] Liu L, Liu J, Huang Z, Yu X, Zhang X, Dou D, et al Berberine improves endothelial function by inhibiting endoplasmic reticulum stress in the carotid arteries of spontaneously hypertensive rats. Biochem Biophys Res Commun. 2015;458(4):796–801. 10.1016/j.bbrc.2015.02.028 . 25686503

[12] Zhang G, Lin X, Shao Y, Su C, Tao J, Liu X. Berberine reduces endothelial injury and arterial stiffness in spontaneously hypertensive rats. Clin Exp Hypertens. 2020;42(3):257–65. 10.1080/10641963.2019.1632339 . 31220947

[13] Wang JM, Yang Z, Xu MG, Chen L, Wang Y, Su C, et al Berberine-induced decline in circulating CD31+/CD42- microparticles is associated with improvement of endothelial function in humans. Eur J Pharmacol. 2009;614(1–3):77–83. 10.1016/j.ejphar.2009.04.037 . 19401197

[14] Zhang H, Niu H, Yuan X, Chang J, Wang X. Trimetazidine combined with berberine on endothelial function of patients with coronary heart disease combined with primary hypertension. Exp Ther Med. 2018;16(2):1318–22. 10.3892/etm.2018.6278 . 30116381 PMC6090239

[15] Li X, Zhang L, Liang J. Unraveling the expression profiles of long noncoding rnas in rat cardiac hypertrophy and functions of lncRNA BC088254 in cardiac hypertrophy induced by transverse aortic constriction. Cardiology. 2016;134(2):84–98. 10.1159/000443370 . 26919297

[16] Wang YN, Shan K, Yao MD, Yao J, Wang JJ, Li X, et al Long noncoding RNA-GAS5: a novel regulator of hypertension-induced vascular remodeling. Hypertension. 2016;68(3):736–48. 10.1161/HYPERTENSIONAHA.116.07259 . 27432865

[17] Dong B, Zhou B, Sun Z, Huang S, Han L, Nie H, et al LncRNA-FENDRR mediates VEGFA to promote the apoptosis of brain microvascular endothelial cells via regulating miR-126 in mice with hypertensive intracerebral hemorrhage. Microcirculation. 2018;25(8):e12499 10.1111/micc.12499 . 30120860

[18] Zhuo X, Wu Y, Yang Y, Gao L, Qiao X, Chen T. LncRNA AK094457 promotes AngII-mediated hypertension and endothelial dysfunction through suppressing of activation of PPARγ. Life Sci. 2019;233:116745 10.1016/j.lfs.2019.116745 . 31404524

[19] Dai W, Mu L, Cui Y, Li Y, Chen P, Xie H, et al Berberine promotes apoptosis of colorectal cancer via regulation of the long non-coding RNA (lncRNA) cancer susceptibility candidate 2 (CASC2)/AU-Binding Factor 1 (AUF1)/B-Cell CLL/Lymphoma 2 (Bcl-2) Axis. Med Sci Monit. 2019;25:730–8. 10.12659/MSM.912082 . 30681073 PMC6357823

[20] Zeng Z, Pan Y, Wu W, Li L, Wu Z, Zhang Y, et al Myocardial hypertrophy is improved with berberine treatment via long non-coding RNA MIAT-mediated autophagy. J Pharm Pharmacol. 2019;71(12):1822–31. 10.1111/jphp.13170 . 31612504

[21] Cao DW, Liu MM, Duan R, Tao YF, Zhou JS, Fang WR, et al The lncRNA Malat1 functions as a ceRNA to contribute to berberine-mediated inhibition of HMGB1 by sponging miR-181c-5p in poststroke inflammation. Acta Pharmacol Sin. 2020;41(1):22–33. 10.1038/s41401-019-0284-y . 31431734 PMC7471439

[22] Huang da W, Sherman BT, Lempicki RA. Systematic and integrative analysis of large gene lists using DAVID bioinformatics resources. Nat Protoc. 2009;4(1):44–57. 10.1038/nprot.2008.211 . 19131956

[23] Yu G, Yao W, Wang J, Ma X, Xiao W, Li H, et al LncRNAs expression signatures of renal clear cell carcinoma revealed by microarray. PloS ONE. 2012;7(8):e42377 10.1371/journal.pone.0042377 . 22879955 PMC3412851

[24] Guo Z, Sun H, Zhang H, Zhang Y. Anti-hypertensive and renoprotective effects of berberine in spontaneously hypertensive rats. Clin Exp Hypertens. 2015;37(4):332–9. 10.3109/10641963.2014.972560 . 25867076

[25] Birney E, Stamatoyannopoulos JA, Dutta A, Guigó R, Gingeras TR, Margulies EH, et al Identification and analysis of functional elements in 1% of the human genome by the ENCODE pilot project. Nature. 2007;447(7146):799–816. 10.1038/nature05874 . 17571346 PMC2212820

[26] Zhang X, Yang X, Lin Y, Suo M, Gong L, Chen J, et al Anti-hypertensive effect of Lycium barbarum L. with down-regulated expression of renal endothelial lncRNA sONE in a rat model of salt-sensitive hypertension. Int J Clin Exp Pathol. 2015;8(6):6981–7. . 26261587 PMC4525921

[27] Fang G, Qi J, Huang L, Zhao X. LncRNA MRAK048635_P1 is critical for vascular smooth muscle cell function and phenotypic switching in essential hypertension. Biosci Rep. 2019;39(3): BSR20182229 10.1042/BSR20182229 . 30833363 PMC6422888

[28] Shi L, Tian C, Sun L, Cao F, Meng Z. The lncRNA TUG1/miR-145-5p/FGF10 regulates proliferation and migration in VSMCs of hypertension. Biochem Biophys Res Commun. 2018;501(3):688–95. 10.1016/j.bbrc.2018.05.049 . 29758198

[29] Yuan X, Wang J, Tang X, Li Y, Xia P, Gao X. Berberine ameliorates nonalcoholic fatty liver disease by a global modulation of hepatic mRNA and lncRNA expression profiles. J Transl Med. 2015;13:24 10.1186/s12967-015-0383-6 . 25623289 PMC4316752

[30] Shreenivas S, Oparil S. The role of endothelin-1 in human hypertension. Clin Hemorheol Microcirc. 2007;37(1–2):157–78. . 17641406

[31] Zhang ML, Zhao GL, Hou Y, Zhong SM, Xu LJ, Li F, et al Rac1 conditional deletion attenuates retinal ganglion cell apoptosis by accelerating autophagic flux in a mouse model of chronic ocular hypertension. Cell Death Dis. 2020;11(9):734 10.1038/s41419-020-02951-7 . 32913260 PMC7484783

[32] Meijles DN, Cull JJ, Markou T, Cooper STE, Haines ZHR, Fuller SJ, et al Redox regulation of cardiac ASK1 (apoptosis signal-regulating kinase 1) controls p38-MAPK (mitogen-activated protein kinase) and orchestrates cardiac remodeling to hypertension. Hypertension. 2020;76(4):1208–18. 10.1161/HYPERTENSIONAHA.119.14556 . 32903101 PMC7480944

[33] Zhang Y, Xu R, Li X, Tan Q, Huang P, Zhang Y, et al Effects of icariin on long noncoding RNA and mRNA expression profile in the aortas of apoE-deficient mice. Biosci Rep. 2019;39(7):BSR20190855 10.1042/BSR20190855 . 31296789 PMC6658818

[34] Pant T, Dhanasekaran A, Bai X, Zhao M, Thorp EB, Forbess JM, et al Genome-wide differential expression profiling of lncRNAs and mRNAs associated with early diabetic cardiomyopathy. Sci Rep. 2019;9(1):15345 10.1038/s41598-019-51872-9 . 31653946 PMC6814824

[35] Liao S, Ou M, Lai L, Lin H, Zou Y, Yu Y, et al Integrated analysis of quantitative proteome and transcriptional profiles reveals abnormal gene expression and signal pathway in bladder cancer. Genes Genomics. 2019;41(12):1493–503. 10.1007/s13258-019-00868-w . 31576517

[36] Pandey KN. Molecular and genetic aspects of guanylyl cyclase natriuretic peptide receptor-A in regulation of blood pressure and renal function. Physiol Genomics. 2018;50(11):913–28. 10.1152/physiolgenomics.00083.2018 . 30169131 PMC6293115

[37] Lin KF, Chao J, Chao L. Human atrial natriuretic peptide gene delivery reduces blood pressure in hypertensive rats. Hypertension. 1995;26(6 Pt 1):847–53. 10.1161/01.hyp.26.6.847 . 7490138

[38] Schillinger KJ, Tsai SY, Taffet GE, Reddy AK, Marian AJ, Entman ML, et al Regulatable atrial natriuretic peptide gene therapy for hypertension. PNAS. 2005;102(39):13789–94. 10.1073/pnas.0506807102 . 16162668 PMC1236585

[39] Suhasini AN, Wang L, Holder KN, Lin AP, Bhatnagar H, Kim SW, et al A phosphodiesterase 4B-dependent interplay between tumor cells and the microenvironment regulates angiogenesis in B-cell lymphoma. Leukemia. 2016;30(3):617–26. 10.1038/leu.2015.302 . 26503641 PMC4775385

[40] Cheng D, Ren J, Gillespie DG, Mi Z, Jackson EK. Regulation of 3’,5’-cAMP in preglomerular smooth muscle and endothelial cells from genetically hypertensive rats. Hypertension. 2010;56(6):1096–101. 10.1161/HYPERTENSIONAHA.110.160176 . 20975032 PMC3011225

[41] Busch CJ, Liu H, Graveline AR, Bloch KD. Nitric oxide induces phosphodiesterase 4B expression in rat pulmonary artery smooth muscle cells. Am J Physiol Lung Cell Mol Physiol. 2006;290(4):L747–l53. 10.1152/ajplung.00298.2005 . 16299053

[42] Ikawa T, Watanabe Y, Okuzaki D, Goto N, Okamura N, Yamanishi K, et al A new approach to identifying hypertension-associated genes in the mesenteric artery of spontaneously hypertensive rats and stroke-prone spontaneously hypertensive rats. J Hypertens. 2019;37(8):1644–56. 10.1097/HJH.0000000000002083 . 30882592 PMC6615961

[43] Duga B, Czako M, Komlosi K, Hadzsiev K, Torok K, Sumegi K, et al Deletion of 4q28.3–31.23 in the background of multiple malformations with pulmonary hypertension. Mol Cytogenet. 2014;7:36 10.1186/1755-8166-7-36 . 24959202 PMC4066825

