# Supplementary material for: Berberine ameliorates vascular dysfunction by a global modulation of lncRNA and mRNA expression profiles in hypertensive mouse aortae
Source: PLoS One. 2021 Feb 23;16(2):e0247621. doi: 10.1371/journal.pone.0247621 (PMC7901729; doi:10.1371/journal.pone.0247621)
Supplement: S1 Table — (DOCX) [file pone.0247621.s001.docx]

S1 Table. The primers of the lncRNAs.

| Gene name |  | Primer sequence (5'-3') |
| --- | --- | --- |
| AK041185 | Forward | CTTCGTTCCTGGAGGCACA |
|  | Reverse | ACAGTCCTGTCTTACACGCACA |
| AK044823 | Forward | TGGGAGTCTGGGTCTCTTGTG |
|  | Reverse | GAGTGGTGCTACCGCTTTCAG |
| AK076651 | Forward | CTCCCACATCTGCTCCACTTT |
|  | Reverse | AGAGGATGCTGTATTCCTTTGTCT |
| BY077582 | Forward | AGCTGAACAACAGGCCAAGA |
|  | Reverse | CCCTTATGGCCGGATCCAAG |
| ENSMUST00000148357 | Forward | TCGGAAAGATTTGGTGGCA |
|  | Reverse | TGACTGGTTACAAGGAGCGGT |
| ENSMUST00000139702 | Forward | CACCTTTCAGGCTGTGAGCTGT |
|  | Reverse | GAAAGAAAGCATGCAGTCATTCAG |
| ENSMUST00000123078 | Forward | TGGACAGCCCACCTTTCTTAG |
|  | Reverse | CTTAACCAGGACTGGAGAAGCA |
| ENSMUST00000072373 | Forward | GAAGGACATGGTGGTGTCTGT |
|  | Reverse | AAAAGGGATGATATGACCAACAGT |
| TCONS_00029108 | Forward | GAGGCTACGGAAGCTCACT |
|  | Reverse | AGCAAGGCGGTACTTTTGTGA |
| NR_028422 | Forward | AATGGTAGAGAGGCACCTGACTG |
|  | Reverse | CAGATTCAGTTCTCTTCAAGGGTG |
| ENSMUST00000161255 | Forward | TCCATCAGTTAATGGGGAGTAGTAG |
|  | Reverse | TTCCTCGATAGACAGCAAAATCC |
| ENSMUST00000161399 | Forward | GATGGTTCGGTGCATGAAGG |
|  | Reverse | TCAGAAGCCAAGCAAGTCCTC |
| ENSMUST00000119528 | Forward | TTGATCCCCATCACAAATGCA |
|  | Reverse | GGATGCCCTTAGTTGTCCAAGG |
| ENSMUST00000155185 | Forward | TCTCTGGACAATGAAGTCTGCC |
|  | Reverse | AGATATGCCCTGACTCTTGCAAA |
| uc007pgi.1 | Forward | GATGAATGTGACCTGGGGAAAG |
|  | Reverse | CAACTGGCTGCTCATGGTGTAC |
| uc011zab.2 | Forward | ACTGCGAGGAGAAGATGGTTATG |
|  | Reverse | TTGTGAGAAAGAAAGGGCTTTTAT |
| ENSMUST00000155383 | Forward | CTGCCTCAGTTTACCCACCAC |
|  | Reverse | GCACAGCAACACCAACTACAGA |
| uc.247+ | Forward | ATAGCGGTATCGATTGATCCTG |
|  | Reverse | AAGCACTGGGCAATAAAACTCA |
| uc.335+ | Forward | AACCACTGGGACCTGAGACC |
|  | Reverse | GGCCATGCATTACTGAACTTG |
| ENSMUST00000144849 | Forward | AAGCAACCAAGAGTCTGGAAACC |
|  | Reverse | ATCATCCCTGGCATCCTGAAC |
| ENSMUST00000147654 | Forward | TCATTCACTTCCTCCTCTACATGCT |
|  | Reverse | TTGCCTAATCCTCTGTAAAGATGCT |
