# Supplementary material for: Berberine ameliorates vascular dysfunction by a global modulation of lncRNA and mRNA expression profiles in hypertensive mouse aortae
Source: PLoS One. 2021 Feb 23;16(2):e0247621. doi: 10.1371/journal.pone.0247621 (PMC7901729; doi:10.1371/journal.pone.0247621)
Supplement: S2 Table — (DOCX) [file pone.0247621.s002.docx]

S2 Table. The primers of the mRNAs.

| Gene name |  | Primer sequence (5'-3') |
| --- | --- | --- |
| *Gapdh* | Forward | GTATGACTCCACTCACGGCAAA |
|  | Reverse | GGTCTCGCTCCTGGAAGATG |
| *Nppa* | Forward | AGAGACGGCAGTGCTCTAGG |
|  | Reverse | GAAGCAGCTGGATCTTCGTA |
| *Hhip* | Forward | TGAAGATGCTCTCGTTTAAGCTG |
|  | Reverse | CCACCACACAGGATCTCTCC |
| *Itga8* | Forward | ATCTCTTGTGCAGTGGGTCG |
|  | Reverse | TTCTTTCTCTTGAGGAACGTGT |
| *Chrm2* | Forward | CGGACCACAAAAATGGCAGGCAT |
|  | Reverse | CCATCACCACCAGGCATGTTGTTGT |
| *Pde4b* | Forward | GACCGGATACAGGTTCTTCG |
|  | Reverse | CAGTGGATGGACAATGTAGTCA |
| *Cdh1* | Forward | CGATTACGAGGGCAGTGGTT |
|  | Reverse | TGTCCGCCAGCTTCTTGAAT |
