# Supplementary material for: Berberine ameliorates vascular dysfunction by a global modulation of lncRNA and mRNA expression profiles in hypertensive mouse aortae
Source: PLoS One. 2021 Feb 23;16(2):e0247621. doi: 10.1371/journal.pone.0247621 (PMC7901729; doi:10.1371/journal.pone.0247621)
Supplement: S3 Table — (DOCX) [file pone.0247621.s003.docx]

S3 Table. Functional annotation of differentially expressed lncRNAs.

| Seqname | GeneSymbol | Source | Chrom | Regulation | Fold Change  （A vs V） | P-value  （A vs V） | Fold Change  (A vs A+B) | P-value  (A vs A+B) |
| --- | --- | --- | --- | --- | --- | --- | --- | --- |
| ENSMUST00000119528 | Gm12054 | Ensembl | chr11 | up | 29.5228344 | 3.11E-05 | 3.4703315 | 5.18E-03 |
| ENSMUST00000161399 | Tnnc1 | Ensembl | chr14 | up | 17.2101568 | 2.33E-05 | 3.4492544 | 2.50E-03 |
| ENSMUST00000155185 | C130080G10Rik | Ensembl | chr2 | up | 12.6081526 | 7.93E-05 | 3.3741589 | 1.92E-03 |
| BY077582 | mouselincRNA1227 | lincRNA | chr5 | up | 10.9953707 | 2.83E-07 | 3.4236469 | 2.56E-02 |
| NR_028422 | C130080G10Rik | RefSeq | chr2 | up | 10.9645753 | 1.06E-04 | 3.4156061 | 2.28E-03 |
| AK044823 | AK044823 | Genbank | chr1 | up | 10.8186042 | 4.61E-03 | 3.1357054 | 8.18E-03 |
| AK076651 | AK076651 | Genbank | chr10 | up | 10.5544266 | 3.50E-03 | 2.894506 | 5.81E-03 |
| AK041185 | AK041185 | Genbank | chr10 | up | 11.4589298 | 2.98E-03 | 3.3392933 | 1.25E-03 |
| ENSMUST00000144849 | 5530601H04Rik | Ensembl | chrX | down | 65.127176 | 8.75E-04 | 32.3679876 | 3.04E-03 |
| uc.335+ | uc.335 | UCR | chr6 | down | 28.6761419 | 2.54E-06 | 7.8086575 | 3.71E-02 |
| ENSMUST00000147654 | 1700047F07Rik | Ensembl | chr4 | down | 18.0768125 | 3.30E-04 | 8.6992973 | 1.41E-03 |
| ENSMUST00000155383 | Daglb | Ensembl | chr5 | down | 13.1631929 | 3.38E-04 | 8.6267166 | 1.20E-03 |
| ENSMUST00000123078 | Ndufaf5 | Ensembl | chr2 | down | 12.3328615 | 2.38E-04 | 5.7963083 | 2.00E-02 |
| TCONS_00029108 | XLOC_021653 | Alexander et al 2013 | chr7 | down | 10.5945627 | 2.06E-03 | 5.9126657 | 5.78E-03 |

Note: V, Vehicle; A, Ang Ⅱ, angiotensin Ⅱ; A+B, Ang Ⅱ+Berberine.
