# Supplementary material for: Berberine ameliorates vascular dysfunction by a global modulation of lncRNA and mRNA expression profiles in hypertensive mouse aortae
Source: PLoS One. 2021 Feb 23;16(2):e0247621. doi: 10.1371/journal.pone.0247621 (PMC7901729; doi:10.1371/journal.pone.0247621)
Supplement: S4 Table — (DOCX) [file pone.0247621.s004.docx]

S4 Table. Functional annotation of differentially expressed mRNAs.

| Seqname | GeneSymbol | Source | Chrom | Regulation | Fold Change  (A vs V) | P-value  (A vs V) | Fold Change  (A vs A+B) | P-value  (A vs A+B) |
| --- | --- | --- | --- | --- | --- | --- | --- | --- |
| NM_008725 | *Nppa* | RefSeq | chr4 | up | 87.4978503 | 3.24E-04 | 27.8280137 | 1.11E-02 |
| NM_203491 | *Chrm2* | RefSeq | chr6 | up | 15.526036 | 7.40E-04 | 7.591075 | 4.72E-02 |
| NM_009864 | *Cdh1* | RefSeq | chr8 | up | 5.2049793 | 8.36E-03 | 5.9470219 | 1.09E-02 |
| NM_001177981 | *Pde4b* | RefSeq | chr4 | down | 3.2122162 | 4.91E-03 | 6.2629577 | 2.56E-02 |
| NM_001001309 | *Itga8* | RefSeq | chr2 | down | 4.755846 | 8.09E-04 | 3.9644004 | 2.11E-03 |
| NM_020259 | *Hhip* | RefSeq | chr8 | down | 4.2083081 | 6.44E-04 | 3.7913063 | 5.09E-05 |

Note: V, Vehicle; A, Ang Ⅱ, angiotensin Ⅱ; A+B, Ang Ⅱ+Berberine.
