# Supplementary material for: Berberine ameliorates vascular dysfunction by a global modulation of lncRNA and mRNA expression profiles in hypertensive mouse aortae
Source: PLoS One. 2021 Feb 23;16(2):e0247621. doi: 10.1371/journal.pone.0247621 (PMC7901729; doi:10.1371/journal.pone.0247621)
Supplement: S1 File — (DOCX) [file pone.0247621.s005.docx]

**Primary culture of mouse aortic endothelial cells (ECs)**

The aortae isolated from mice were placed in Dulbecco's modified Eagle medium (DMEM), cleaned of adhering tissues, and then digested by 0.2% collagenase type IA for 8 min at 37°C. After digestion, DMEM with 10% fetal bovine serum (FBS) was added. By centrifugation at1000 rpm for 5 minutes, detached cells were collected. Next, the supernatant was moved and the pellet was re-suspended in DMEM with 20% FBS and incubated in cell culture dish at 37°C. After 45 minutes, the medium was changed by EGM-2 medium (Lonza, Walkersville, MD) with 10% FBS.

**Primary culture of mouse aortic smooth muscle cells (SMCs)**

After cleaning of adhering tissue, aortae were cut longitudinally, scraped off endothelium, and then placed in a 60 mm cell culture dish containing 2 ml DMEM with 10% FBS. Next, the aortae were cut into pieces about 1mm×1mm and then dispersed in a 35mm cell culture dish. After the pieces adhered firmly to the bottom of the dish, 2 ml DMEM with 20% FBS were slowly added. The cells crawled out from the tissue blocks after 2-3 days and maintained until 80% confluence before use.

.
