# Supplementary figures and images for: Berberine ameliorates vascular dysfunction by a global modulation of lncRNA and mRNA expression profiles in hypertensive mouse aortae
Source: PLoS One. 2021 Feb 23;16(2):e0247621. doi: 10.1371/journal.pone.0247621 (PMC7901729; doi:10.1371/journal.pone.0247621)

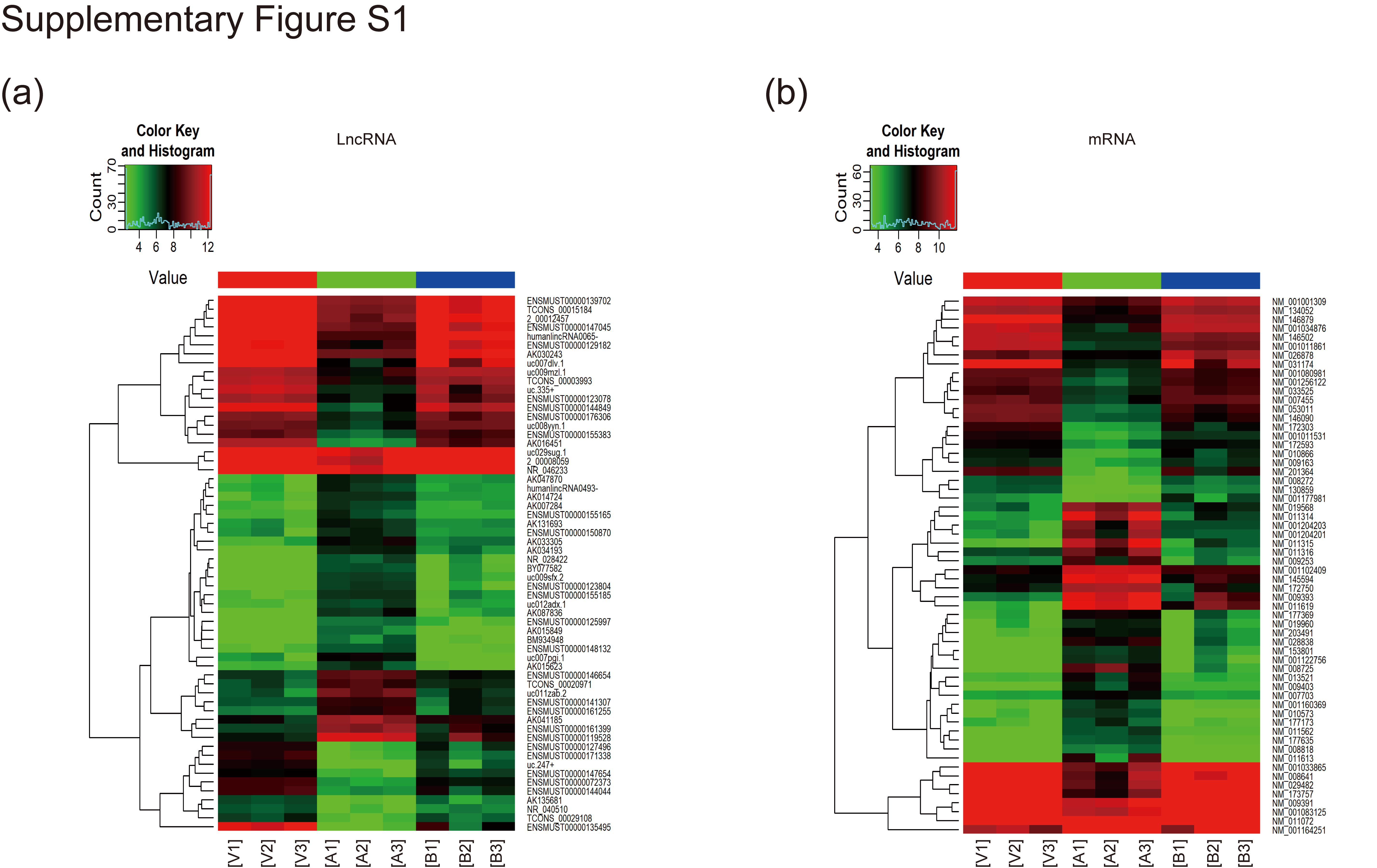

Supplement: S1 Fig — It showed top 60 differentially expressed lncRNAs (a) and mRNAs (b) among the three groups. Red color indicates highly relative expression and green color indicates low relative expression. V, Vehicle; A, Ang Ⅱ, angiotensin Ⅱ; B, Ang Ⅱ+Berberine. (TIF) [file pone.0247621.s006.tif]

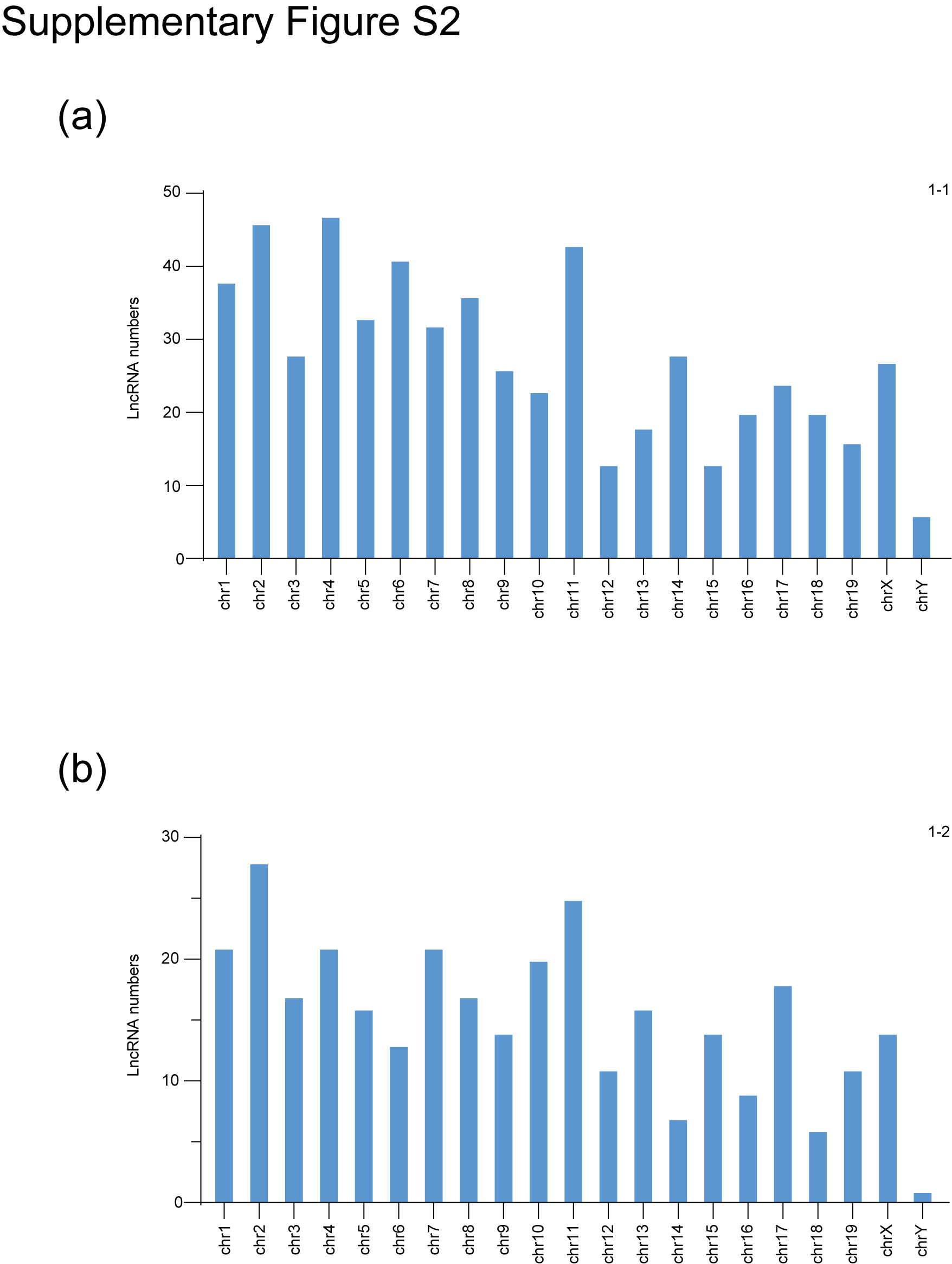

Supplement: S2 Fig — Chromosomal distribution of the 1–1 (a) and 1–2 (b) lncRNAs. 1–1, genes up-regulated by Ang Ⅱ but down-regulated by co-treatment with berberine; 1–2, genes suppressed by Ang Ⅱ while reversed by berberine. Ang Ⅱ, angiotensin Ⅱ. (TIF) [file pone.0247621.s007.tif]

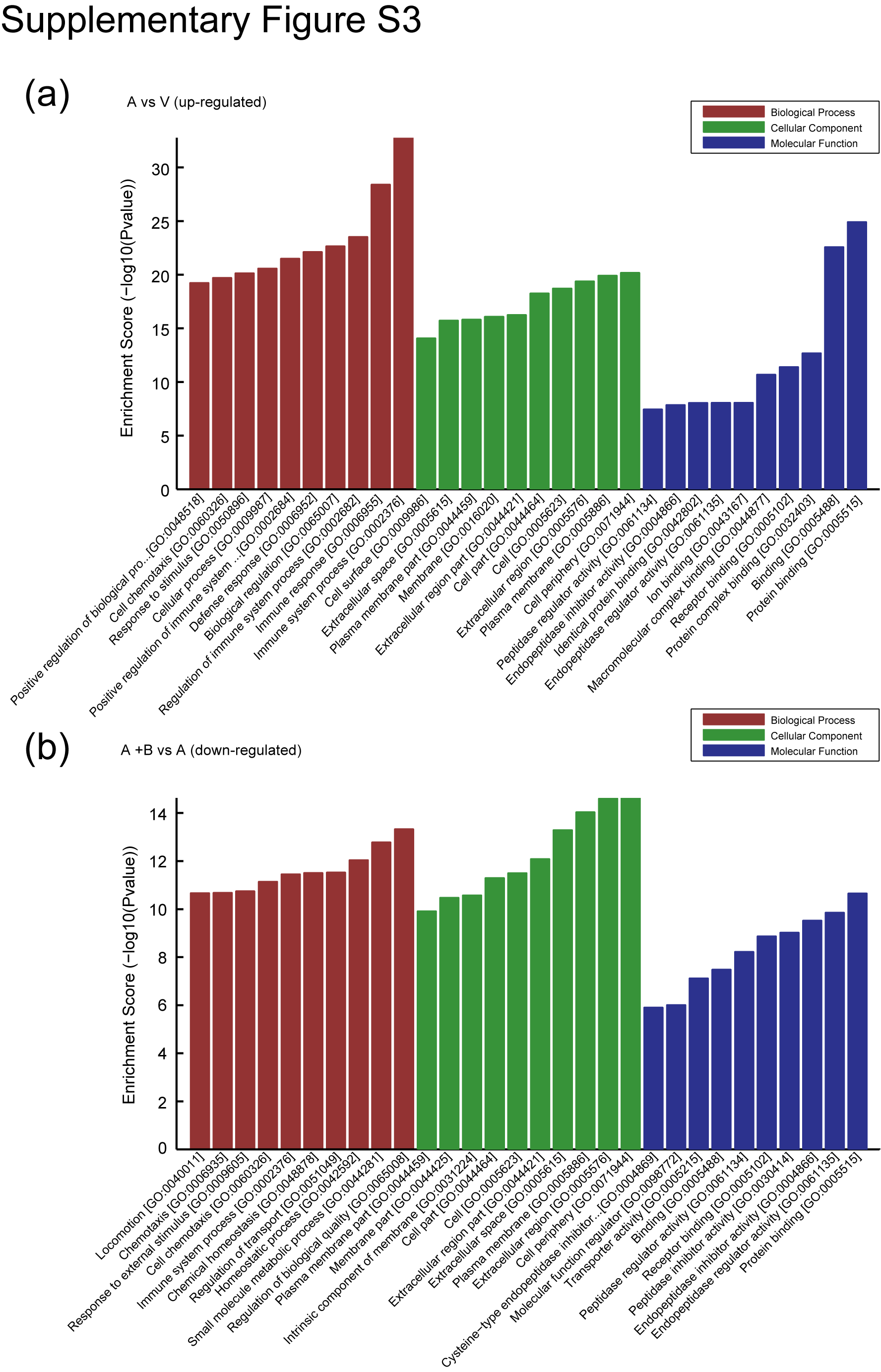

Supplement: S3 Fig — Red, green and blue bars represented biological process (BP), cellular component (CC), and molecular function (MF). (a) The top 10 GO terms that were associated with the coding gene function of up-regulated lncRNAs in the Ang Ⅱ-treated group compared with Vehicle-treated group. (b) the top 10 GO terms that were associated with the coding gene function of down-regulated lncRNAs in the Ang Ⅱ+Berberine co-treated group compared with Ang Ⅱ-treated group. 1–1, genes up-regulated by Ang Ⅱ but down-regulated by berberine; V, Vehicle; A, Ang Ⅱ, angiotensin Ⅱ; A+B, Ang Ⅱ+Berberine. (TIF) [file pone.0247621.s008.tif]

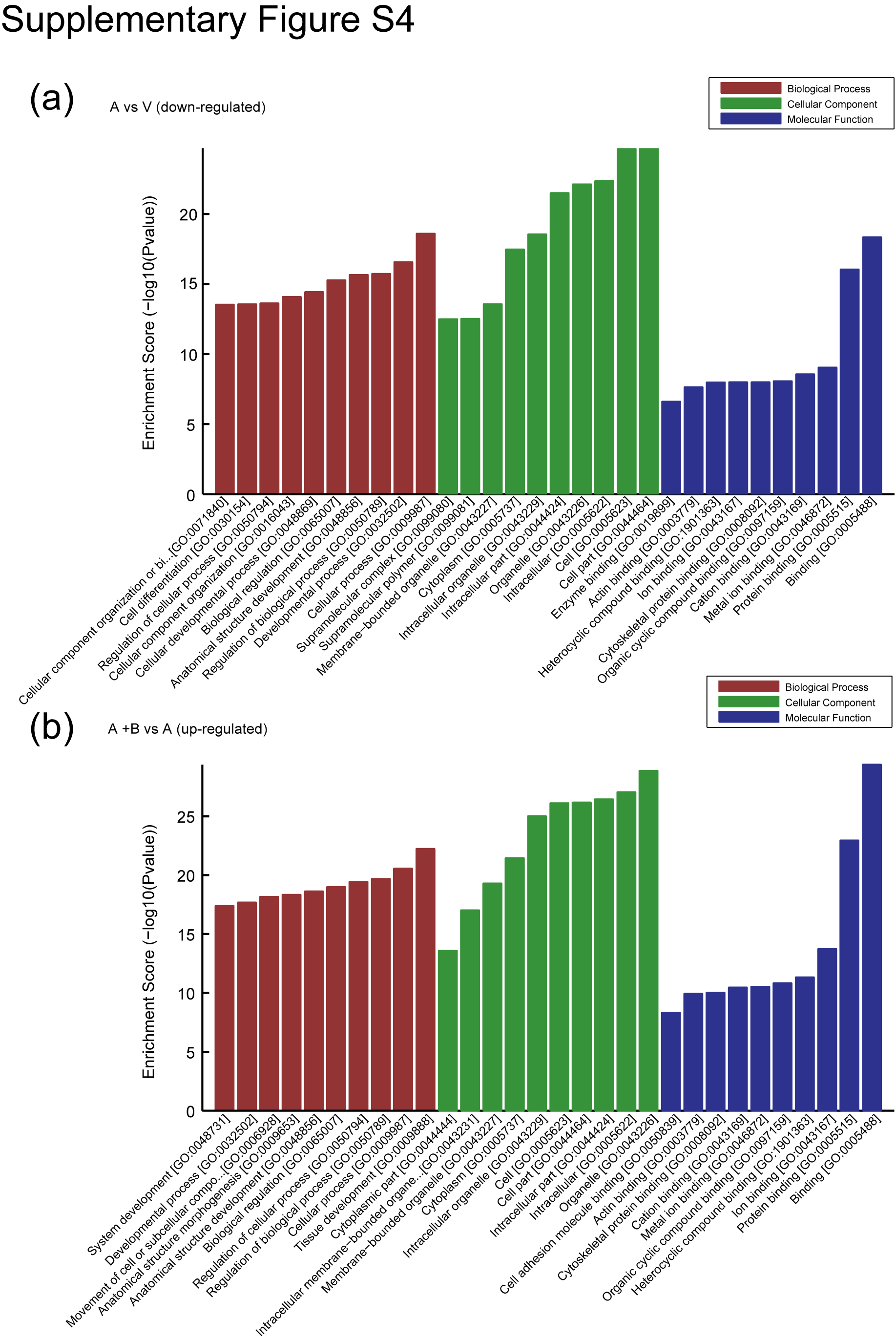

Supplement: S4 Fig — Red, green and blue bars represented biological process (BP), cellular component (CC), and molecular function (MF). (a) The top 10 GO terms that were associated with the coding gene function of down-regulated lncRNAs in the Ang Ⅱ-treated group compared with Vehicle-treated group. (b) the top 10 GO terms that were associated with the coding gene function of up-regulated lncRNAs in the Ang Ⅱ+Berberine co-treated group compared with Ang Ⅱ-treated group. 1–2, genes suppressed by Ang Ⅱ while increased by berberine; V, Vehicle; A, Ang Ⅱ, angiotensin Ⅱ; A+B, Ang Ⅱ+Berberine. (TIF) [file pone.0247621.s009.tif]

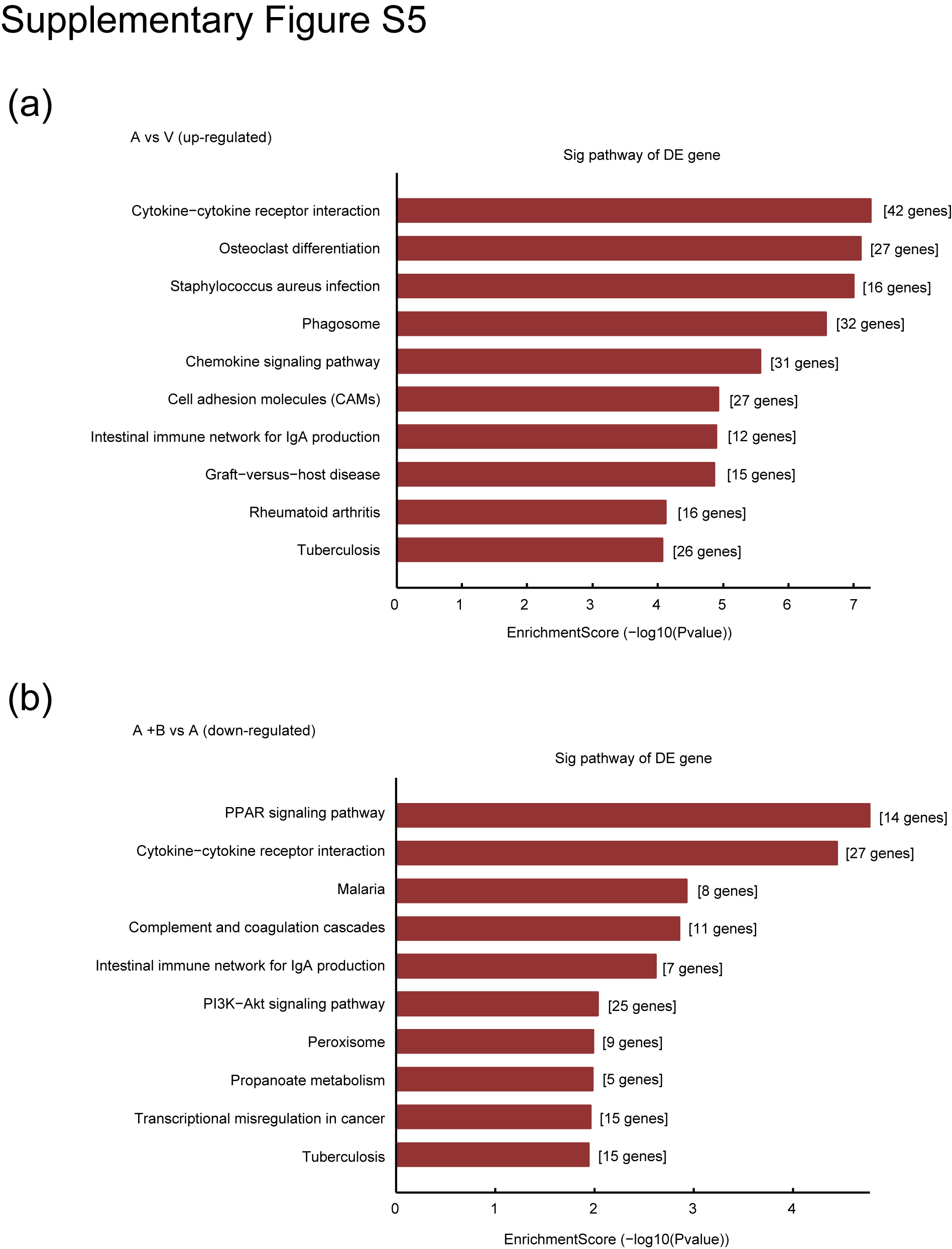

Supplement: S5 Fig — It revealed that (a) the top 10 pathways that were associated with the coding gene function of up-regulated lncRNAs in the Ang Ⅱ-treated group compared with Vehicle-treated group, (b) the top 10 pathways that were associated with the coding gene function of down-regulated lncRNAs in the Ang Ⅱ+Berberine co-treated group compared with Ang Ⅱ-treated group. 1–1, genes up-regulated by Ang Ⅱ but down-regulated by berberine; V, Vehicle; A, Ang Ⅱ, angiotensin Ⅱ; A+B, Ang Ⅱ+Berberine. (TIF) [file pone.0247621.s010.tif]

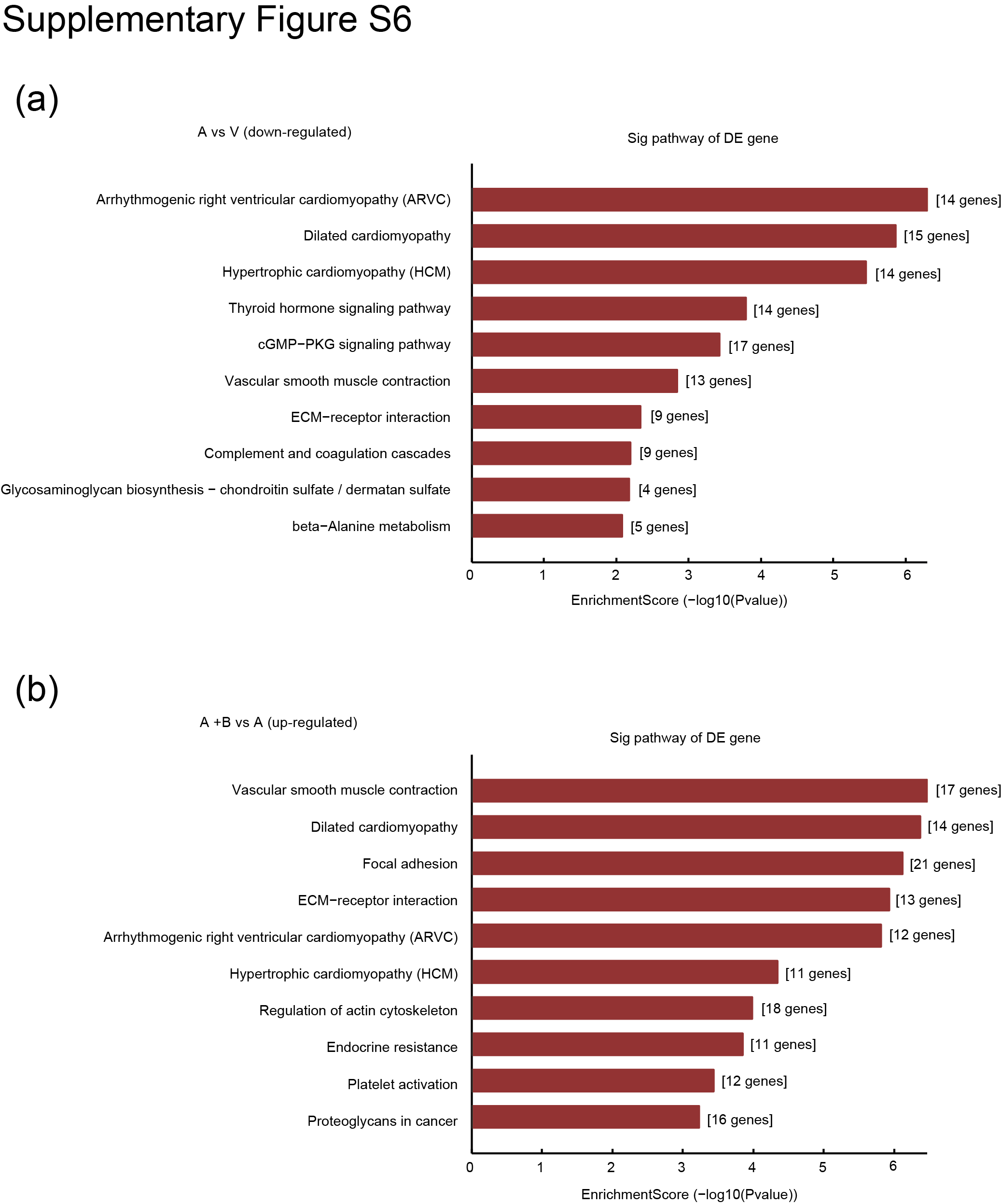

Supplement: S6 Fig — It showed (a) the top 10 pathways that were associated with the coding gene function of down-regulated lncRNAs in the Ang Ⅱ-treated group compared with Vehicle-treated group, (b) the top 10 pathways that were associated with the coding gene function of up-regulated lncRNAs in the Ang Ⅱ+Berberine co-treated group compared with Ang Ⅱ-treated group. 1–2, genes suppressed by Ang Ⅱ while increased by berberine; V, Vehicle; A, Ang Ⅱ, angiotensin Ⅱ; A+B, Ang Ⅱ+Berberine. (PNG) [file pone.0247621.s011.png]

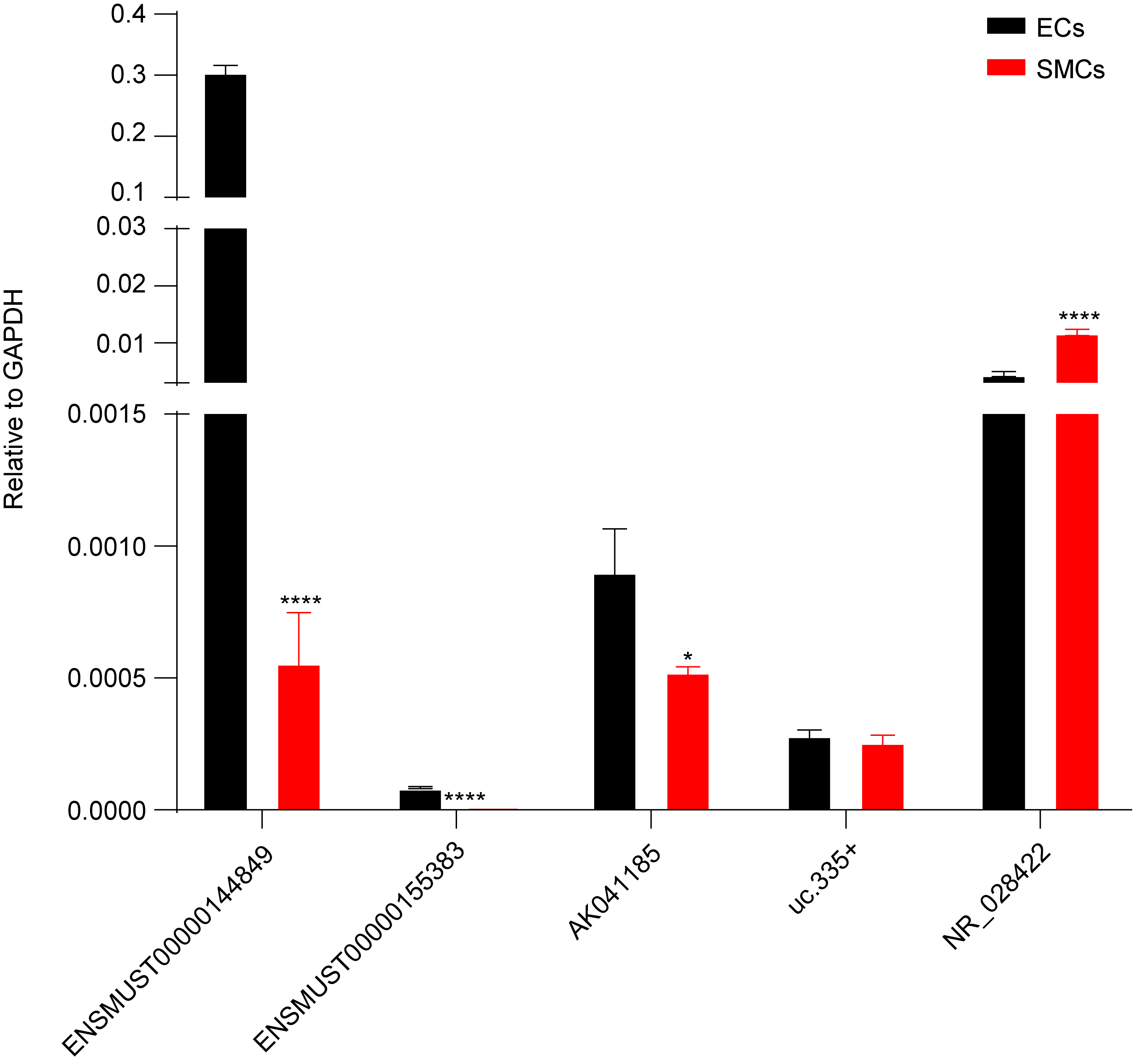

Supplement: S7 Fig — LncRNA ENSMUST00000144849, lncRNA ENSMUST00000155383, lncRNA AK041185, and lncRNA NR_028422 exhibited different expression levels in ECs and SMCs; but lncRNA uc.335+ expression was similar in the two cell types. EC, endothelial cells; SMCs, smooth muscle cells. (TIF) [file pone.0247621.s012.tif]
