# Supplementary material for: Berberine ameliorates vascular dysfunction by a global modulation of lncRNA and mRNA expression profiles in hypertensive mouse aortae
Source: PLoS One. 2021 Feb 23;16(2):e0247621. doi: 10.1371/journal.pone.0247621 (PMC7901729; doi:10.1371/journal.pone.0247621)
Supplement: Attachment — Submitted filename: Response to Reviewers.docx [file pone.0247621.s013.docx]

The authors thank the constructive comments and suggestions of the two reviewers and we respond point-by-point as follows. We performed some new experiments to support our conclusion and also made necessary discussion.

**Response to Reviewers' Comments:**

**Reviewer 1**

Na Tan et al performed the microarray and qRT-PCR to show the regulatory effects of berberine on the expression profiles of lncRNA and mRNAs in hypertensive mouse aortae. The experiments provide significant and novel results about the molecular mechanisms of berberine in hypertension; and the manuscript are well written. I only have a few minor suggestions.

**Authors’ response:** We thank this reviewer for the very positive appraisal of our findings and manuscript.

1. More information is necessary for the animal protocol:
1) What is the dosage of Ang Ⅱ osmotic pumps implanted in the mice?

**Authors’ response:** The dosage of Ang Ⅱ is 1mg/kg/day. We revised with the detail in Materials and Methods section (please see page 4, line 106 in the revised manuscript) as “Ang II (1mg/kg/day)- or PBS-loaded osmotic pumps were implanted in mice”.

2) What are sources and the solvents for Ang Ⅱ and berberine?

**Authors’ response:** Ang Ⅱ and berberine chloride were respectively purchased from Tocris Bioscience (Bristol, UK) and Sigma-Aldrich Chemical (St Louis, MO, USA). We revised with the detail in Materials and Methods section (please see page 4, lines 110-112 in the revised manuscript) as “Ang II and berberine chloride were respectively purchased from Tocris Bioscience (Bristol, UK) and Sigma-Aldrich Chemical (St Louis, MO, USA). Ang II was dissolved in PBS. Berberine was dissolved in drinking water”.

3) Is berberine administered by oral gavage or other method?

**Authors’ response:** The mice received berberine administration (100 mg/kg/day) or vehicle in drinking water. We revised with the detail in Materials and Methods section (please see page 4, line 108 in the revised manuscript) as “Then the mice received berberine administration (100 mg/kg/day) or vehicle in drinking water for 2 weeks.”

2. The authors can mention the limitations of current study in the discussion part. There are correlation about the changes of expression profiles of lncRNA and mRNAs with the berberine treatment in hypertension; nevertheless, it is not sure whether such changes lead to the improvement of endothelial function. Further investigations are needed to prove that the changes lead to the endothelial dysfunction in hypertension and vaso-protective effects of berberine.

**Authors’ response:** We thank this reviewer for the constructive suggestion. We discussed the limitations in the revised manuscript (Page 12, lines 465-477).

**Reviewer 2**

This study mainly investigated the potential mechanism underlying the therapeutic effects of berberine against Ang II-induced hypertension. By using microarray technology, the authors revealed that in Ang II group, 578 lncRNAs and 554 mRNAs were up-regulated, 320 lncRNAs and 377 mRNAs were downregulated in the aortae, while some of them were reversed by berberine treatment. By deep analysis of these differentially expressed (DE) genes, they found that these DE genes were closely associated with NO production and vascular tone regulation. Based on these findings, the authors proposed 5 potential LncRNAs that may participate in the blood pressure-lowering effect of berberine. In general, this study is well designed and written. However, I have several concerns.

**Authors’ response:** We thank this reviewer for the positive appraisal of our findings and manuscript.

1. Is there any study showing the association between the 5 LncRNAs with blood pressure regulation?

**Authors’ response:** Till now, there is no evidence to state the association between the 5 LncRNAs with blood pressure regulation. But we will further explore the regulatory effects of these 5 lncRNAs on endothelial function and blood pressure in hypertension in future study. In addition, we discussed this limitation in the revised manuscript (Page 12, lines 465-477).

2. Are the 5 LncRNAs majorly expressed in endothelial cells?

**Authors’ response:** In order to explain this point, we primarily cultured endothelial cells (ECs) and smooth muscle cells (SMCs) of the aortae from C57/6J mice. We performed qRT-PCR to examine the expressions of the 5 lncRNAs both in ECs and in SMCs. As shown in S7 Fig or in the figure below, the levels of lncRNA ENSMUST00000155383, AK041185, and ENSMUST00000144849 were significantly higher in ECs than those in SMCs. LncRNA uc.335+ and lncRNA NR_028422 were also clearly expressed in ECs; but the expression level of lncRNA uc.335+ was similar in ECs and SMCs and the expression level of lncRNA NR_028422 in SMCs was higher than that in ECs. Moreover, we discussed this in the revised manuscript (Page 11, lines 446-455).
